# Structural Embedding of Oral Health Within Pooled Universal Coverage Mechanisms: Where Are We in 2026?

**DOI:** 10.3390/healthcare14081104

**Published:** 2026-04-20

**Authors:** Carol Moussa, Marion Mégly, Jeremy Glomet, Maha H. Daou, Céline Clement, Frédéric Denis

**Affiliations:** 1 Division of Education, Ethics, Health, Faculty of Medicine, University of Tours, 37044 Tours, France; celine.clement@univ-tours.fr (C.C.); frederic.denis@univ-tours.fr (F.D.); 2 Faculty of Dentistry, University of Tours, 37032 Tours, France; maha.daou@univ-tours.fr; 3 Department of Medicine and Bucco-Dental Surgery, Tours University Hospital, 37044 Tours, France; marion.megly@univ-tours.fr (M.M.); jeremy.glomet@univ-tours.fr (J.G.)

**Keywords:** oral health, universal health care, health policy, public health, delivery of health care, global health, World Health Organization

## Abstract

Oral diseases affect an estimated 3.5 billion people globally and remain among the most prevalent noncommunicable conditions. Despite recent global policy commitments under the WHO Global Oral Health Strategy and Action Plan (2023–2030), substantial variation persists in how countries structurally embed oral health within national health systems. A structural classification of all 194 WHO Member States was conducted using WHO 2022 oral health country profiles and official policy documentation. Countries were categorized according to financing architecture and entitlement design into four integration models: Structural UHC Integration, Partial or Targeted Integration, Predominantly Private or Insurance-Driven Systems, and Minimal or Emerging Integration. Regional and global distributions were calculated using RStudio (version 2025.09.0, Posit Software, PBC, Boston, MA, USA). Globally, Partial or Targeted Integration represents the most common configuration (44%), followed by Predominantly Private systems (17%) and Minimal or Emerging Integration (15%), while Structural UHC Integration accounts for approximately 10% of countries. Marked regional heterogeneity was observed, with Structural UHC Integration concentrated in selected regions and Minimal or Emerging models more prevalent in parts of Africa and South-East Asia. Findings suggest that integration is primarily determined by financing architecture and legally defined entitlements rather than national income level alone. Structural embedding of oral health within pooled universal coverage mechanisms appears to be an important structural feature associated with higher levels of integration.

## 1. Introduction

Oral diseases affect an estimated 3.5 billion people globally and remain among the most prevalent noncommunicable conditions worldwide [1]. Dental caries, periodontal disease, and tooth loss contribute substantially to pain, functional impairment, reduced quality of life, and economic burden across all income settings [2,3,4]. Despite this widespread impact, oral health has historically occupied a peripheral position within national health systems and universal health coverage (UHC) frameworks.

Oral diseases share common risk factors with other major noncommunicable diseases (NCDs), including tobacco use, unhealthy diets, and socioeconomic disadvantage [5,6,7]. Yet, unlike many other NCDs, dental services are frequently excluded from publicly financed benefit packages or restricted to specific population groups, resulting in high out-of-pocket expenditure and persistent inequities in access [8,9,10].

Recent global policy developments, including the World Health Assembly resolution on oral health (WHA74.5) and the WHO Global Strategy and Action Plan for Oral Health (2023–2030), have called for integration of essential oral health services into primary health care and publicly financed benefit packages [11,12,13]. Despite these commitments, substantial variation persists in how countries structurally organize and finance oral healthcare.

Existing analyses have primarily examined epidemiological trends or national case studies. Fewer studies have examined oral health integration from a comparative structural perspective across WHO Member States. In particular, there remains a gap between global policy ambitions and comparative system-level understanding of the financing and entitlement arrangements that shape integration in practice.

To address this gap, the present study develops a descriptive global classification of oral health system integration models across WHO Member States based on financing architecture and entitlement design. Countries were categorized into four structural models: Structural UHC Integration, Partial or Targeted Integration, Predominantly Private or Insurance-Driven Systems, and Minimal or Emerging Integration. The aim was to provide a comparative framework for understanding how oral health is positioned within national health systems worldwide.

## 2. Background: Global WHO Framework for Oral Health

The contemporary global governance framework for oral health is anchored in a sequence of World Health Assembly resolutions and strategic instruments adopted between 2021 and 2024. Together, these documents establish a policy architecture intended to reposition oral health within universal health coverage (UHC) and noncommunicable disease (NCD) agendas.

WHA74.5 (2021) formally recognized oral health as a global public health priority and called for stronger integration of oral health into national NCD strategies and health systems [9]. Building on this mandate, the Global Strategy on Oral Health, endorsed in 2022 (WHA75.11), articulated a vision of universal access to essential oral health services by 2030 and emphasized integration into primary health care and publicly financed benefit packages [10].

The Global Oral Health Action Plan 2023–2030 (WHA76.9, 2023) operationalizes this vision through defined strategic domains, including governance, prevention, workforce development, service delivery, information systems, and research, and measurable global targets [11]. Prominent objectives include expanding population entitlement to essential oral health services under UHC frameworks and reducing the global burden of major oral diseases relative to 2020 baselines. The Action Plan also encourages workforce planning, sugar reduction policies, fluoride delivery strategies, integration of oral health into primary care, and alignment with environmental sustainability commitments such as the Minamata Convention [11,12].

Complementary analytical instruments, notably the Global Oral Health Status Report, provide baseline data and highlight persistent inequalities in disease burden, service access, and financial protection [13]. High out-of-pocket expenditures, limited public financing, workforce constraints, and weak integration into general health services were identified as structural barriers to progress [13].

Momentum for implementation was reinforced at the first WHO Global Oral Health Meeting in Bangkok in 2024, where the Bangkok Declaration reaffirmed oral health as a political priority within UHC and NCD frameworks and called for accelerated national action to embed oral health within primary care systems and publicly financed benefit packages [14,15].

Collectively, these instruments constitute a coherent global framework against which national oral health system integration can be assessed. They provide normative benchmarks for evaluating the extent to which countries have structurally embedded oral health within financing architecture, entitlement design, and primary health care systems.

## 3. Methods

### 3.1. Study Design

This study conducts a descriptive cross-sectional structural classification of oral health system integration models and provides descriptive contextualization using selected country examples to illustrate contrasting implementation pathways.

### 3.2. Primary Data Source

The principal data source for structural classification was the WHO 2022 Oral Health Country Profiles, which provide standardized information on governance, financing, service delivery, and surveillance. These data were complemented, when necessary, by official national policy government publications (e.g., Ministry of Health documents, statutory insurance descriptions) and intergovernmental health system sources (e.g., OECD, World Bank, European Observatory).

A targeted grey literature search was conducted to clarify missing or ambiguous information on financing mechanisms and entitlement structures. This search was not systematic but focused on official and authoritative sources.

Data collection was performed between October 2025 and February 2026.

### 3.3. Unit of Analysis

The unit of analysis was the WHO Member State. All 194 Member States were screened for structural characteristics of oral health integration within national health systems.

### 3.4. Data Extraction and Abstraction Process

Data extraction was conducted independently by two reviewers (C.M. and C.C.) using a standardized data abstraction framework developed for this study.

Extracted variables included: financing mechanism (tax-based, social insurance, mixed, private), population entitlement to oral health services, scope of publicly financed services (preventive, essential, advanced), degree of integration within primary healthcare, and reliance on out-of-pocket expenditure.

Discrepancies between reviewers were resolved through consensus discussion, and when necessary, adjudicated by a third senior author (F.D.).

### 3.5. Structural Classification Framework

Countries were classified using a hierarchical decision rule. Where the WHO 2022 Oral Health Country Profile explicitly reported oral health inclusion within national health benefit packages and/or provided coverage percentages for the largest government financing scheme, classification was based directly on these structural indicators.

In cases where the WHO benefit package matrix was incomplete or not reported, and where official national documentation did not clearly confirm pooled dental entitlement, countries were conservatively retained as “No Data” to avoid misclassification.

Countries were categorized according to dominant financing architecture and entitlement framework governing access to oral health services. Four structural models were defined a priori:

1. **Structural UHC Integration:**

Oral health services embedded within pooled universal health coverage mechanisms with defined population entitlements.

2. **Partial or Targeted Integration:**

Publicly financed oral health services limited to specific population groups (e.g., children, low-income groups) or restricted benefit packages.

3. **Predominantly Private or Insurance-Driven Systems:**

Dental services largely financed through private insurance or out-of-pocket payments, with limited pooled public coverage.

4. **Minimal or Emerging Integration:**

Limited public provision, weak financing structures, and constrained infrastructure for oral health service delivery.

Where WHO profile information was insufficient to determine structural positioning with confidence, countries were designated as “No Data.”

The classification aimed to reflect the predominant system logic, acknowledging that many countries operate mixed or hybrid models.

### 3.6. Classification Criteria

Classification was based on structural indicators including:

‑ Breadth of entitlement to essential oral health services
‑ Financing mechanism (tax-based, social insurance, mixed, or private)
‑ Degree of integration within primary healthcare
‑ Reliance on out-of-pocket expenditure

Structural classification was anchored in the WHO 2022 Oral Health Country Profiles. When WHO benefit-package fields were incomplete or ambiguous, classification was refined using official national sources describing dental entitlement and financing architecture (e.g., statutory insurance scope, government benefit packages, national health insurance documentation), supplemented where necessary by intergovernmental health system references (e.g., OECD/World Bank/European Observatory) that explicitly report dental coverage arrangements. Countries were labeled ‘No Data’ only when WHO profile information was missing and no reliable official documentation could be identified. Classification focused on dominant system architecture rather than isolated programs.

Classification followed a sequential decision rule according to the following operational decision algorithm:

1. Countries were classified as **Structural UHC Integration** when preventive and essential oral health services were included in publicly financed benefit packages covering a majority of the population (operationalized as ≥50%).
2. Countries were classified as **Partial or Targeted Integration** when public coverage was restricted to specific population groups (e.g., children, low-income populations) or limited service packages.
3. Countries were classified as **Predominantly Private** when dental services were mainly financed through out-of-pocket payments or private insurance.
4. Countries were classified as **Minimal or Emerging Integration** when public provision was limited, fragmented, or restricted to emergency care.

The ≥50% threshold was used as an operational criterion to define majority population coverage and standardize classification across countries. This threshold does not imply a normative benchmark and is acknowledged as a methodological simplification.

### 3.7. Handling of Missing Data

When WHO profiles and supporting documentation did not provide sufficient information to determine classification with confidence, countries were categorized as “No Data.”

This conservative approach was adopted to avoid misclassification. However, it may introduce bias, particularly affecting low- and middle-income countries with limited publicly available documentation.

### 3.8. Income-Level and Regional Classification

Countries were grouped according to the official World Health Organization (WHO) regional classification (AFRO, AMRO, EMRO, EURO, SEARO, WPRO). Income-level categorization was based on the World Bank country income classification for the fiscal year 2024, which classifies countries as low-income, lower-middle-income, upper-middle-income, or high-income economies based on gross national income per capita. These variables were used for descriptive analysis only and not for causal inference.

### 3.9. Data Analysis

Global and regional distributions of structural integration models were calculated descriptively using RStudio (version 2025.09.0, Posit Software, PBC, Boston, MA, USA).

Results are presented as frequencies and percentages across WHO Member States.

No inferential statistical analyses were performed.

### 3.10. Narrative Contextualization

Selected countries were included for illustrative purposes to highlight variation in implementation pathways.

These examples were selected purposively based on geographic diversity, income level, and availability of documentation

This approach does not aim to provide a representative sample and may introduce illustrative bias, which is acknowledged in the limitations.

The study protocol is registered on the Open Science Framework (OSF). All classification decisions and data abstraction procedures followed predefined criteria to enhance reproducibility.

## 4. Results

### 4.1. Global Structural Classification of WHO Member States

**Figure 1:**
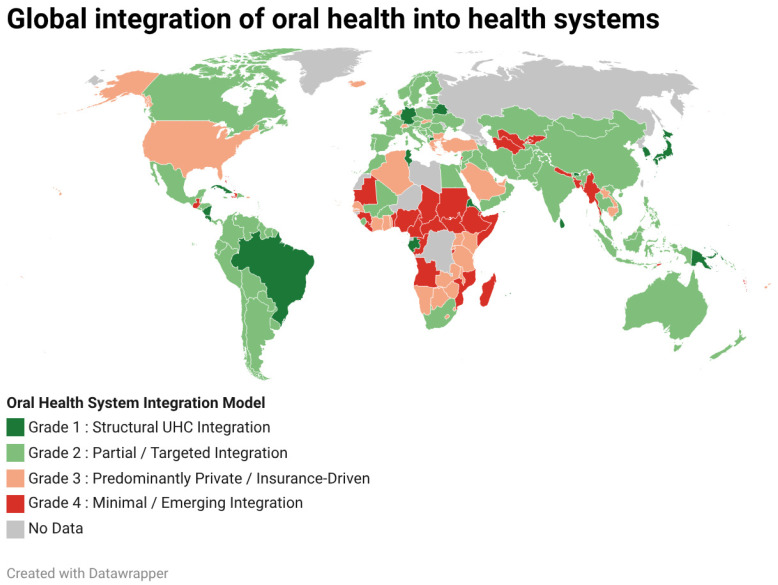
Global integration of oral health into health system integration models across WHO Member States.

Figure 1 presents a global classification of oral health system integration models based on the current structural organization of dental care within national health systems. Countries were categorized according to the dominant financing and entitlement framework governing access to oral health services.

Classification reflects whether dental care is embedded within pooled universal health coverage mechanisms (Structural UHC Integration), provided through partial or population-targeted public schemes (Partial or Targeted Integration), primarily reliant on private insurance or out-of-pocket financing (Predominantly Private or Insurance-Driven Systems), or characterized by limited public provision and constrained infrastructure (Minimal or Emerging Integration).

In cases where publicly available information was insufficient to determine the structural model with confidence, countries were designated as “No Data”. Supplementary Table S1 includes the structural classification for all 194 WHO Member States. Classification was determined based on publicly available policy documentation and WHO country profiles, assessing entitlement breadth, financing mechanisms, and degree of integration within primary health care systems.

Supplementary Table S1 includes structural classification for all 194 WHO Member States.

Classification is based on WHO 2022 Oral Health Country Profiles and complementary official national sources. Countries were categorized according to dominant financing architecture and entitlement design. The classification reflects structural organization and does not assess performance or outcomes.

Across WHO Member States, structural integration models demonstrate substantial heterogeneity. Globally, Structural UHC Integration accounts for approximately 10% of countries, while Partial or Targeted Integration represents the most common model at 44%. Predominantly Private or Insurance-Driven systems comprise 17%, and Minimal or Emerging Integration accounts for 15% of Member States. Countries classified as “No Data” represent approximately 14%, reflecting limitations in publicly available structural documentation.

Regional variation is pronounced. Structural UHC Integration is most prevalent in the Western Pacific Region (21%) and the South-East Asia Region (18%), while Partial or Targeted Integration predominates in the Region of the Americas (77%) and the Eastern Mediterranean Region (59%). Predominantly Private systems are most frequently observed in the African Region (38%) and parts of the Western Pacific (21%). Minimal or Emerging Integration is concentrated in the African Region (31%) and South-East Asia (36.5%), underscoring persistent structural financing and infrastructure constraints in several low- and middle-income settings. Regional and global distributions are presented in Supplementary Table S2.

These findings highlight substantial global and regional heterogeneity in the structural organization of oral health systems. In particular, Partial or Targeted Integration emerges as the dominant model worldwide, while comprehensive structural integration within universal coverage remains relatively limited.

### 4.2. Regional Distribution Patterns

Marked regional variation was observed in the distribution of structural integration models.

In the Western Pacific and South-East Asia regions, Structural UHC Integration was more frequently identified compared to other regions, although it remained a minority configuration overall. In contrast, Partial or Targeted Integration predominated in the Americas and Eastern Mediterranean regions, representing the most common structural arrangement.

Predominantly Private systems were more frequently observed in parts of Africa and the Western Pacific, while Minimal or Emerging Integration was concentrated in Africa and South-East Asia, reflecting more limited structural embedding of oral health within publicly financed systems.

These regional differences suggest that structural integration varies according to broader health system organization and policy trajectories, rather than following a uniform global pattern. In particular, regions characterized by stronger public financing mechanisms tended to show higher levels of structural integration, although this relationship was not systematic across all countries.

### 4.3. Association with Income Level and System Organization (Descriptive Analysis)

No consistent relationship was observed between national income level and structural integration model.

While several high-income countries demonstrated Structural UHC Integration, others were classified as Predominantly Private or Partial Integration systems, indicating substantial variability within income groups. Conversely, some middle-income countries exhibited higher levels of structural integration through publicly financed primary care approaches.

Overall, Partial or Targeted Integration remained the most frequent configuration across all income groups, suggesting that full structural integration is not solely determined by economic capacity.

These findings indicate that financing architecture and entitlement design may play a more important role than income level alone in shaping integration models. However, these observations are descriptive and should not be interpreted as causal relationships.

### 4.4. Comparative Patterns in Policy and Implementation (Narrative Synthesis)

National oral health policies vary widely in scope, maturity, and degree of integration into health systems. High-income countries generally have long-established dental care infrastructures and some form of national guidance or insurance coverage, although the emphasis on prevention and equity differs substantially. In contrast, many low- and middle-income countries (LMICs) have only recently developed comprehensive oral health policies, often in response to the WHO Global Strategy and Action Plan [16].

Across settings, most national policies address common objectives: improving oral hygiene practices, preventing dental caries and periodontal disease, and expanding access to basic dental treatment. However, differences emerge in the extent of public coverage, the strength of population-level preventive measures, and the integration of oral health into primary care and universal health coverage (UHC) [16].

To illustrate variation in implementation pathways, selected country examples are presented in Supplementary Table S3 [13,17,18,19,20,21,22,23,24,25,26,27,28,29,30,31,32,33,34,35,36,37,38,39,40,41,42,43,44,45,46,47,48,49,50,51,52,53,54,55,56,57,58,59,60,61,62,63,64,65,66,67,68,69,70,71,72,73,74,75,76]. The country examples included in this table were selected purposively to reflect diversity in income level, geographic region, health system organization, and stages of oral health policy development. The aim was not to provide an exhaustive global inventory, but to illustrate contrasting policy models and implementation pathways within different health system contexts. Countries were chosen based on the availability of official policy documentation, relevance to the WHO Global Oral Health Strategy, and their representativeness of distinct approaches to governance, financing, prevention, and integration of oral health into primary healthcare and universal health coverage.

Across countries, several recurring structural patterns were identified:

‑ Systems with pooled public financing and defined entitlements tended to include preventive and basic oral health services within primary care.
‑ Systems with mixed or targeted coverage often restricted access to specific population groups, particularly children.
‑ Systems relying predominantly on private financing showed greater dependence on out-of-pocket expenditure and more limited population coverage.
‑ In settings with minimal integration, services were frequently limited to emergency or hospital-based care

These patterns reflect differences in governance, financing models, and policy prioritization across countries.

## 5. Cross-Cutting Structural Determinants of Integration

### 5.1. Integrating Oral Health into Primary Care and Universal Health Coverage (UHC)

From a macroeconomic perspective, oral health expenditure represents a non-negligible share of health system resources. In high-income settings, spending on oral healthcare is estimated to account for approximately 0.5–1.0% of gross domestic product (GDP) and 5–10% of total health expenditure, highlighting the substantial economic footprint of oral diseases and dental care [77,78]. These aggregate estimates align with the financing patterns summarized in Supplementary Table S3.

Across many countries, oral healthcare remains characterized by a high reliance on direct household payments, with a substantial proportion of dental expenditure often financed through out-of-pocket (OOP) spending, which contributes to delayed care, unmet needs, and socioeconomic gradients in utilization [13,78].

In low- and middle-income countries (LMICs), available estimates indicate that dental expenditure is generally lower (often <0.3% of GDP), reflecting chronic underinvestment, limited preventive service delivery, and a persistent dependence on private payment mechanisms rather than pooled financing [13].

Beyond financing levels, legal and regulatory frameworks play a decisive role in determining whether oral health is effectively integrated into primary healthcare and UHC. In several countries, integration has been underpinned by binding legislation or statutory insurance frameworks. Thailand’s National Health Security Act established a legal basis for including essential dental services within the Universal Coverage Scheme [79], while successive revisions of Japan’s Health Insurance Act explicitly embedded comprehensive dental care within universal social health insurance [80]. In other contexts, integration has proceeded through policy instruments and regulatory reforms rather than stand-alone dental laws, as illustrated by Kenya’s National Oral Health Policy (2022–2030) [81] and Saudi Arabia’s Health Sector Transformation Program under Vision 2030 [40], which incorporate oral health within broader primary care, prevention, and noncommunicable disease strategies.

Conversely, the absence of binding legislation has contributed to fragmented implementation in several high-income settings. Switzerland, for example, lacks a federal legal framework integrating dental care into mandatory health insurance, resulting in decentralized, canton-based arrangements and continued reliance on out-of-pocket payment despite overall strong health system performance [82]. Taken together, these experiences indicate that adequate financing alone is insufficient: sustained integration of oral health into primary care and UHC is more likely when supported by explicit legal or regulatory mandates that define entitlements, financing mechanisms, and accountability structures [83].

Integration of oral health into primary healthcare (PHC) and universal health coverage (UHC) is a central pillar of the WHO Global Oral Health Strategy and a recurring objective across national reforms. Integration implies that essential preventive and basic curative oral health services are delivered alongside other primary care services, financed through pooled funding mechanisms, and accessible without prohibitive financial barriers. When effectively implemented, this approach improves access, supports early prevention, and reduces avoidable complications and catastrophic out-of-pocket expenditure [84].

Despite broad political endorsement, implementation remains uneven. In many countries, routine dental care is excluded from UHC benefit packages or limited to specific population groups, most commonly children [2,3]. As a result, dental services continue to operate largely outside mainstream health systems, particularly for adults. High out-of-pocket costs discourage timely care, leading to late presentation, tooth loss, and preventable hospital admissions for dental infections [6,85,86,87]. In low-resource settings, public oral health services are often confined to urban hospitals, with primary-level facilities lacking dental personnel, equipment, or referral pathways [88,89].

Countries that have advanced integration provide instructive contrasts. Thailand’s Universal Coverage Scheme has included preventive and basic dental services for more than two decades, delivered through an extensive primary care network. This integration has been associated with high utilization rates and relatively low levels of untreated disease among children [65,66]. Brazil similarly embedded oral health within its Family Health Strategy, deploying multidisciplinary teams that include oral health professionals at community level [33,90]. These models demonstrate that integration is feasible when supported by sustained public financing, workforce planning, and strong governance.

High-income countries have pursued more incremental pathways. Sweden and other Nordic countries provide publicly funded dental care for children and adolescents, combined with subsidies for adults, resulting in high coverage and low unmet need [48,88,91]. France has expanded coverage for essential dental services through recent insurance reforms while strengthening preventive pathways across the life course [53,54,55,56]. Australia and Canada, historically characterized by limited public dental coverage, have introduced targeted benefits for children and vulnerable adults as entry points toward broader integration [92,93,94].

Beyond financing, service delivery models are central to integration. Several countries have expanded school-based dental programs, mobile clinics, and community outreach as mechanisms to deliver primary oral healthcare in underserved areas [88,95]. Task-sharing approaches (training nurses, community health workers, or mid-level dental providers to deliver basic preventive services) have been adopted in settings with dentist shortages [89,96]. Teledentistry has also emerged as a complementary strategy, particularly in geographically remote regions, enabling remote consultation, triage, and supervision [97].

Integration gaps are evident even within healthcare institutions. Studies consistently show deterioration of oral hygiene during hospitalization, reflecting limited protocols, insufficient staff training, and the persistent separation of oral care from routine medical practice. These findings underscore that integration requires system-wide change, including clinical guidelines, workforce competencies, and accountability mechanisms, rather than reliance on individual behavior alone [8].

Overall, integration into PHC and UHC should be understood as a continuum. Countries that have made progress typically began with defined essential service packages, priority population groups, and incremental workforce expansion. The WHO target that 80% of the population be entitled to essential oral health services by 2030 has catalyzed policy dialogue [12].

### 5.2. Prevention-Oriented Strategies

Prevention lies at the core of contemporary oral health policy and represents the most effective and cost-efficient approach to reducing the global burden of oral disease [98,99]. Dental caries and periodontal diseases are largely preventable through well-established interventions targeting shared risk factors, notably high sugar consumption, tobacco use, inadequate fluoride exposure, and suboptimal oral hygiene practices [3,88,100]. Consistent with the WHO Global Oral Health Strategy, prevention-oriented policies increasingly adopt a common risk factor approach, aligning oral health with broader noncommunicable disease (NCD) prevention agendas [11,101].

**Population-Level Prevention and Common Risk Factor Approaches:** Prevention-oriented oral health policies increasingly focus on population-level interventions targeting shared risk factors, including fluoride exposure, sugar consumption, tobacco use, and harmful alcohol intake [2,100]. Rather than isolated dental measures, these approaches emphasize alignment with broader nutrition and noncommunicable disease prevention policies, reflecting a shift toward intersectoral action as the foundation for sustainable oral health improvement [98,101].

**Individual and Clinical Prevention Across the Life Course:** At the individual level, daily oral hygiene practices remain fundamental. Many countries have institutionalized oral health education through school-based programs, often combined with supervised toothbrushing, fluoride rinses, or sealant application [102]. Such programs have demonstrated effectiveness in reducing caries risk, particularly in disadvantaged populations [103]. Community health workers and primary care providers increasingly play a role in delivering oral health education and preventive advice, especially for high-risk groups such as young children, pregnant women, and individuals with chronic diseases [89,98]. Clinical prevention has also expanded through minimal intervention dentistry and risk-based care, with fluoride varnish and fissure sealants integrated into routine pathways, representing a scalable opportunity for population-level impact [104,105,106].

### 5.3. Oral Healthcare Financing and Coverage Reforms

Financing arrangements are a central determinant of access to oral healthcare and a key driver of inequalities in oral health outcomes. Historically, dental care has been one of the most privatized components of health systems worldwide, with a large share of expenditure borne directly by individuals [2,6]. High out-of-pocket payments contribute to delayed care, unmet need, and preventable complications, reinforcing social gradients in oral health [47,98]. In recent years, however, oral healthcare financing has increasingly been recognized as integral to universal health coverage (UHC) and financial risk protection [2,50].

**Tax-funded national health systems:** In tax-funded systems, such as those operating in the United Kingdom, the Nordic countries, and parts of Southern Europe, dental care is financed primarily through general government revenue and delivered through public services or contracted providers. These models aim to ensure broad population coverage, particularly for children and vulnerable groups, and are generally associated with lower financial barriers to access compared with predominantly private systems [56]. Nevertheless, fiscal constraints can limit workforce capacity and access; in England, for example, funding pressures and contractual arrangements have constrained adult access to NHS dentistry despite universal entitlement [98,107]. These experiences underscore that financing design must be accompanied by adequate investment and incentive structures that support prevention-oriented care [98].

**Social health insurance models:** Social health insurance systems integrate dental care into mandatory insurance schemes financed through payroll contributions or premiums, often supplemented by public subsidies [108]. Countries such as Japan, Germany, and France provide near-universal dental coverage through SHI or mixed models, typically covering preventive and basic restorative care with regulated co-payments. Japan exemplifies extensive integration of dental care within SHI, fostering regular attendance and early intervention [73,109], while Germany combines routine coverage with optional supplementary insurance [110]. France’s recent 100% Santé reform further expanded financial protection by eliminating out-of-pocket payments for a defined basket of essential prosthetic services [54,55]. Despite challenges related to benefit design and sustainability, SHI models illustrate how broad risk pooling can reduce financial barriers and improve equity when oral health is explicitly included [98].

**Predominantly private and out-of-pocket systems:** In systems where dental care is largely financed privately, access is closely linked to income and insurance status, particularly for adults [2,92]. High out-of-pocket costs contribute to delayed or forgone care, resulting in higher levels of untreated disease and preventable emergency service use [86,87]. Public provision is often limited to children or emergency treatment, offering limited protection against financial hardship [98]. In the United States, recent analyses have further highlighted how gaps in dental insurance coverage intersect with structural racism, contributing to persistent racial and ethnic inequities in oral health outcomes and access to care [111]. Recognition of these shortcomings has prompted ongoing policy debates and shifts regarding the expansion of comprehensive adult dental benefits within public insurance programs [93,94].

**Financing reforms and future directions:** Across income settings, a common trend is the gradual expansion of publicly financed oral health services, often beginning with priority populations such as children, older adults, or low-income groups [2,98]. Defining essential oral health benefit packages that emphasize prevention and primary care has emerged as a pragmatic entry point for integration into UHC [10,100]. Evidence increasingly supports the cost-effectiveness of such investments, including reductions in emergency department visits and hospital admissions for preventable dental conditions [86,112]. For low-income countries, incremental inclusion of basic oral health services within national benefit packages, aligned with broader UHC reforms, offers a sustainable pathway toward equity [113]. Overall, financing reform is indispensable to achieving equitable oral health outcomes [6,47] and translating WHO 2030 commitments into tangible health gains [9].

## 6. Discussion

This study provides a global descriptive classification of how oral health is structurally embedded within national health systems, based on financing architecture and entitlement design. The findings highlight substantial heterogeneity across WHO Member States, with most countries relying on partial, targeted, or predominantly private models rather than comprehensive structural integration within universal coverage frameworks.

The findings reveal a marked divergence between global policy commitments and structural implementation across national contexts. The WHO Global Oral Health Strategy and Action Plan have established a coherent international framework centered on prevention, primary care integration, and financial protection [9,10,11]. However, structural positioning varies substantially between countries, reflecting differences in financing architecture, governance capacity, and institutional design. This variation underscores that policy ambition alone does not ensure systemic integration; institutional embedding within financing mechanisms and entitlement law appears to play a central role. Integration of oral health into primary healthcare and universal health coverage is consistently observed as a key structural feature across more integrated systems, as identified in this classification. Countries that have incorporated oral health within pooled public financing mechanisms and primary care networks demonstrate more comprehensive population coverage and reduced barriers to access. Experiences from Brazil [2,33] and Thailand [66,67] illustrate how embedding oral health within publicly financed primary care systems can expand access and address unmet need. In contrast, systems that rely predominantly on private financing or out-of-pocket payment, even in high-income settings, tend to exhibit persistent inequalities and delayed care utilization [98,111]. These observations should be interpreted as descriptive associations rather than causal relationships, given the cross-sectional design of the study.

These patterns suggest that structural integration may be associated with entitlement design rather than with national income level alone [47,98]. However, this interpretation should be made cautiously, as the present study does not allow for causal inference.

Prevention-oriented strategies constitute another essential dimension of integration. Population-level measures, including fluoride delivery systems, sugar reduction policies, and tobacco control, offer substantial potential for sustainable impact when embedded within broader public health frameworks [2,98]. However, implementation remains heterogeneous across countries and may depend on broader system-level factors beyond financing structures alone.

Financing reform remains central to addressing structural inequities. Systems that pool risk and prioritize essential preventive and restorative services within publicly financed frameworks demonstrate more consistent access and financial protection [2,6]. Nevertheless, the present analysis does not evaluate outcomes directly, and therefore cannot determine the effectiveness of specific financing models. Incremental expansion of publicly financed benefits—frequently beginning with children or vulnerable populations—has emerged as a common pathway toward broader integration within UHC systems [10,11]. However, financing architecture alone does not guarantee effective delivery. Sustainable integration requires concurrent investment in workforce capacity, service organization, accountability mechanisms, and clinical governance structures [3,98,107]. Observations of inadequate oral health management in institutional and hospital settings further illustrate that integration must extend beyond insurance coverage to include operational protocols and institutional responsibility [8,114,115].

Governance capacity consistently differentiates sustained integration from fragmented implementation. The existence of a national oral health policy does not in itself ensure systemic embedding. Countries demonstrating stable progress typically feature dedicated leadership within ministries of health, integration of oral health indicators into national monitoring systems, and alignment between financing mechanisms and service delivery models [10,11,99]. However, these observations remain descriptive and may be influenced by contextual factors not captured in this analysis. Conversely, fragmented governance structures may limit continuity and reduce the prioritization of oral health within broader health system agendas [100].

Important documentation gaps persist in certain regions, including parts of Eastern Europe and West Africa, where publicly accessible information on financing, entitlement, and surveillance systems remains limited. This limitation may introduce **systematic bias**, particularly affecting low- and middle-income countries, and should be considered when interpreting global patterns. Structural classification reflects implemented entitlement frameworks and financing architecture at the time of analysis. While several countries are engaged in reform trajectories, structural positioning is based on operationalized financing and legally defined entitlements rather than announced policy intentions. Canada represents a clear example of transitional expansion, with phased implementation of federally financed adult dental benefits progressively broadening population coverage [29,30,31]. In contrast, reforms in countries such as France and Germany primarily involve benefit refinement within established social insurance frameworks, without altering core financing architecture [48,49,53,54,55,110]. In Saudi Arabia and the United Arab Emirates, broader health system transformation initiatives may influence future integration depending on the extent to which dental services become embedded within pooled entitlement mechanisms [38,39,40]. In Australia and the United States, ongoing policy discussions regarding adult dental coverage have yet to result in structural integration shifts [23,24,28,94,111]. Continued monitoring will therefore be essential as reforms mature within evolving UHC strategies.

Overall, the findings indicate that structural embedding of oral health within pooled financing and legally defined entitlement is consistently associated with higher levels of integration. However, given the descriptive nature of this analysis, this relationship should not be interpreted as causal. Systems that institutionalize oral health within universal coverage architecture seem to establish the structural foundation necessary for equitable access. In contrast, reliance on fragmented or predominantly private financing models constrains integration, irrespective of economic development. Progress toward the WHO 2030 objectives will depend not only on policy commitments but on sustained institutional reform that aligns financing, governance, prevention, and service delivery within coherent health system architecture.

## 7. Limitations

This study has several limitations that should be considered when interpreting the findings. First, the structural classification relied primarily on publicly available policy documentation, including WHO country profiles and official government sources. While these materials provide standardized and authoritative information, documentation may not fully capture subnational variation, informal service arrangements, or recent unpublished reforms. Countries with limited publicly accessible information were classified as “No Data” to avoid speculative assignment. This approach, while conservative, may lead to underrepresentation of certain health system configurations, particularly in contexts where documentation is limited.

Second, structural categorization reflects implemented entitlement frameworks and financing architecture at the time of analysis. In contexts where reforms have been recently announced or are being phased in, there may be a temporal lag between policy adoption and full operational implementation. The analysis is cross-sectional and does not capture dynamic changes over time, including ongoing reforms or policy transitions.

Third, in federated or highly decentralized health systems, including countries such as Canada, the United States, Australia, and Germany, oral health financing and service delivery may vary across provinces, states, or regions. Although classification was based on dominant national-level entitlement frameworks, subnational heterogeneity may influence access patterns and benefit design in practice.

The structural classification framework involves a degree of interpretative judgment. Although standardized criteria and a predefined decision algorithm were used, the classification of countries based on “dominant system architecture” may oversimplify complex and hybrid health systems. In addition, the operational threshold used to define majority population coverage (≥50%) represents a pragmatic choice to ensure comparability across countries and does not constitute a normative benchmark.

The designation of countries as “No Data” may introduce systematic bias, as missing or incomplete information is more likely to affect low- and middle-income countries. As a result, global distribution patterns may be influenced by differential data availability rather than true underlying system characteristics.

The analysis is conducted at the country level and does not account for within-country variation. Consequently, the findings may be subject to ecological fallacy, and interpretations should not be extended to individual-level access, service utilization, or clinical outcomes.

Finally, this analysis focuses on structural integration defined by financing architecture and entitlement design. It does not directly measure service quality, workforce capacity, or clinical outcomes. Therefore, the classification should be interpreted as a representation of system organization rather than an evaluation of effectiveness or performance.

## 8. Conclusions

The present analysis demonstrates that oral health system integration varies markedly across WHO Member States and reflects progressive stages of structural embedding within national health financing architecture. Countries in which oral health is incorporated into pooled universal coverage mechanisms tend to exhibit the highest level of structural integration, while systems predominantly reliant on private financing or limited public provision remain structurally constrained in their organization. The WHO Global Strategy and Action Plan provide a coherent framework for advancing integration, emphasizing entitlement expansion, primary care embedding, workforce development, and preventive policy alignment. However, the findings of this study suggest that implementation remains heterogeneous across countries and is influenced by differences in financing structures and institutional organization. Structural embedding of oral health within pooled financing mechanisms appears to be an important structural feature associated with higher levels of integration, but this relationship should be interpreted cautiously given the descriptive nature of the analysis. Further research is needed to examine how these structural configurations relate to health outcomes, access to care, and system performance.

## Data Availability

All data used in this study were derived from publicly available sources, including WHO 2022 Oral Health Country Profiles and official national policy documentation. The structured classification dataset generated during the analysis is available from the corresponding author upon reasonable request.

## Supplementary Materials

The following supporting information can be downloaded at: https://www.mdpi.com/article/10.3390/healthcare14081104/s1, Table S1: Structural Classification of Oral Health System Integration Across WHO Member States, Table S2: Regional Distribution of Structural Integration Models. Table S3: Comparison of national oral health policies and implementation across selected countries.

## Abbreviations

The following abbreviations are used in this manuscript:

AFRO	WHO African Region
AMRO	WHO Region of the Americas
EMRO	WHO Eastern Mediterranean Region
EURO	WHO European Region
SEARO	WHO South-East Asia Region
WPRO	WHO Western Pacific Region
WHO	World Health Organization
WHA	World Health Assembly
UHC	Universal Health Coverage
NCDs	Noncommunicable Diseases
PHC	Primary Health Care
OOP	Out-of-Pocket
GDP	Gross Domestic Product
SHI	Social Health Insurance
NHS	National Health Service
UCS	Universal Coverage Scheme
COHI	Child Oral Health Initiative
ECC	Early Childhood Caries
SES	Socioeconomic Status
NHANES	National Health and Nutrition Examination Survey

## References

[1] World Health Organization (2022). WHO Highlights Oral Health Neglect Affecting Nearly Half of the World’s Population.

[2] Peres M.A., Macpherson L.M.D., Weyant R.J., Daly B., Venturelli R., Mathur M.R., Listl S., Celeste R.K., Guarnizo-Herreño C.C., Kearns C. (2019). Oral Diseases: A Global Public Health Challenge. Lancet.

[3] Watt R.G., Aida J. (2022). Time to Take Oral Health Seriously. Lancet Healthy Longev..

[4] Țica O., Romanul I., Ciavoi G., Pantea V.A., Scrobota I., Șipoș L., Daina C.M., Țica O. (2025). A Clinical Review of the Connections Between Diabetes Mellitus, Periodontal Disease, and Cardiovascular Pathologies. Biomedicines.

[5] Jain N., Dutt U., Radenkov I., Jain S. (2024). WHO’s Global Oral Health Status Report 2022: Actions, Discussion and Implementation. Oral Dis..

[6] Listl S., Galloway J., Mossey P.A., Marcenes W. (2015). Global Economic Impact of Dental Diseases. J. Dent. Res..

[7] Jevdjevic M., Listl S. (2025). Global, Regional, and Country-Level Economic Impacts of Oral Conditions in 2019. J. Dent. Res..

[8] Kassem A.O., Umer M.F., Hamidaddin M.A., Nasir E.F., Alomran A.J., Alsuwayi H.I., AlQahtani M.A., Mahabob Basha N., Bokhari S.A.H. (2025). Oral Hygiene Practices of Hospitalized Patients in Public and Private Hospitals in Al-Ahsa, Saudi Arabia: A Cross-Sectional Study. J. Clin. Med..

[9] World Health Organization (2021). Oral Health: Resolution WHA74.5.

[10] World Health Organization (2022). Global Strategy on Oral Health.

[11] World Health Organization (2023). Global Oral Health Action Plan 2023–2030.

[12] Rendell N. Global Oral Health Action Plan 2023–2030 and Monitoring Framework. Proceedings of the FDI Chief Dental Officers/Dental Public Health Section Business Meeting.

[13] World Health Organization (2022). Global Oral Health Status Report: Towards Universal Health Coverage for Oral Health by 2030.

[14] Bangkok Declaration—No Health Without Oral Health. https://www.who.int/publications/m/item/bangkok-declaration---no-health-without-oral-health.

[15] Samaranayake L., Phantumvanit P., Varenne B. (2025). Bangkok Declaration on Oral Health: A Clarion Call for Action by All Stakeholders. Int. Dent. J..

[16] Oral Health Surveys: Basic Methods—5th Edition.

[17] World Health Organization (2022). Seychelles: Oral Health Country Profile.

[18] World Health Organization (2022). South Africa: Oral Health Country Profile.

[19] van Wyk P.J., van Wyk C. (2004). Oral Health in South Africa. Int. Dent. J..

[20] World Health Organization (2022). Kenya: Oral Health Country Profile.

[21] Chen J., Duangthip D., Gao S.S., Huang F., Anthonappa R., Oliveira B.H., Turton B., Durward C., El Tantawi M., Attia D. (2021). Oral Health Policies to Tackle the Burden of Early Childhood Caries: A Review of 14 Countries/Regions. Front. Oral Health.

[22] World Health Organization (2022). Uganda: Oral Health Country Profile.

[23] Northridge M.E., Kumar A., Kaur R. (2020). Disparities in Access to Oral Health Care. Annu. Rev. Public Health.

[24] Oral Health in America: Advances and Challenges|NIDCR. https://www.nidcr.nih.gov/research/oralhealthinamerica.

[25] Fellows J.L., Atchison K.A., Chaffin J., Chávez E.M., Tinanoff N. (2022). Oral Health in America: Implications for Dental Practice. J. Am. Dent. Assoc..

[26] Adesanya M.R., Bailey W., Belcher D.C., Beltran M., Branch T., Brand M.K., Craft E.M., Donahue A.H., Dye B.A., U.S. Department of Health and Human Services Oral Health Coordinating Committee (2016). U.S. Department of Health and Human Services Oral Health Strategic Framework, 2014–2017. Public Health Rep..

[27] Quiñonez C., Jones J.A., Vujicic M., Tomar S.L., Lee J.Y. (2022). The 2021 Report on Oral Health in America: Directions for the Future of Dental Public Health and the Oral Health Care System. J. Public Health Dent..

[28] Gupta N., Vujicic M., Yarbrough C., Harrison B. (2018). Disparities in Untreated Caries among Children and Adults in the U.S., 2011–2014. BMC Oral Health.

[29] Cheung A., Singhal S. (2023). Towards Equitable Dental Care in Canada: Lessons from the Inception of Medicare. Int. J. Health Plann. Manag..

[30] Allison P.J. (2023). Canada’s Oral Health and Dental Care Inequalities and the Canadian Dental Care Plan. Can. J. Public Health.

[31] Rock L.D., Akade G., Al-Waeli H., Allin S., Altabtbaei K., Ameli N., Bassim C., Bedos C., Benbow P., Bhagirath A.Y. (2025). Canada’s First National Oral Health Research Strategy (2024–2030). J. Dent. Res..

[32] Hermosillo V.H., Quintero L.E., Guerrero N.D., Suárez D.D.S., Hernández M.J.A., Holmgren C.J. (2009). The Implementation and Preliminary Evaluation of an ART Strategy in Mexico: A Country Example. J. Appl. Oral Sci..

[33] Pucca G.A., Gabriel M., de Araujo M.E., de Almeida F.C.S. (2015). Ten Years of a National Oral Health Policy in Brazil: Innovation, Boldness, and Numerous Challenges. J. Dent. Res..

[34] Santos L.P.d.S., Lima A.M.F.d.S., Chaves S.C.L., Vilela D.M.O.C., Valente A.P.P.C., Rossi T.R.A. (2023). Oral Health Policy in Brazil: Changes and Ruptures during the Period 2018–2021. Ciênc. Saúde Coletiva.

[35] Brazil’s National Oral Health Policy: An Example for Other Nations. https://www.sciencedaily.com/releases/2015/08/150827154505.htm.

[36] World Health Organization (2022). Oral Health Country Profile: Haiti.

[37] Elamin A., Garemo M., Gardner A. (2018). Dental Caries and Their Association with Socioeconomic Characteristics, Oral Hygiene Practices and Eating Habits among Preschool Children in Abu Dhabi, United Arab Emirates—The NOPLAS Project. BMC Oral Health.

[38] Ministry of Health and Prevention (2018). National Strategy for Wellbeing 2031.

[39] World Health Organization (2022). Regional Office for the Eastern Mediterranean Noncommunicable Diseases Country Profile: United Arab Emirates.

[40] Ministry of Health (2021). Health Sector Transformation Program: Saudi Vision 2030.

[41] Famurewa B.A., Aborisade A.O., Dabar A.M., Akinsolu F.T., El Tantawi M., Ezechi O.C., Foláyan M.O. (2025). Prevalence and Risk Factors for Early Childhood Caries in North Africa: A Systematic Review and Meta-Analysis. BMC Oral Health.

[42] World Health Organization (2019). Libya: Health System Review.

[43] Leggett H., Vinall-Collier K., Csikar J., Veronica Ann Douglas G. (2023). Barriers to Prevention in Oral Health Care for English NHS Dental Patients: A Qualitative Study of Views from Key Stakeholders. BMC Oral Health.

[44] Evans D., Mills I., Burns L., Bryce M., Hanks S. (2023). The Dental Workforce Recruitment and Retention Crisis in the UK. Br. Dent. J..

[45] McAuliffe Ú., Eaton K., Harding M., Whelton H., Cronin J., Burke S. (2025). ‘At a Tipping Point’: A Comparative Analysis of Oral Health Coverage for Children across Six European Countries: Denmark, Germany, Hungary, Ireland, Scotland, and Spain. BMC Oral Health.

[46] (2020). Evaluation of a National Complex Oral Health Improvement Programme: A Population Data Linkage Cohort Study in Scotland. BMJ Open.

[47] Palència L., Espelt A., Cornejo-Ovalle M., Borrell C. (2014). Socioeconomic Inequalities in the Use of Dental Care Services in Europe: What Is the Role of Public Coverage?. Community Dent. Oral Epidemiol..

[48] Ziller S., Eaton K.E., Widström E. (2015). The Healthcare System and the Provision of Oral Healthcare in European Union Member States. Part 1: Germany. Br. Dent. J..

[49] Nomura M. (2008). Dental Healthcare Reforms in Germany and Japan: A Comparison of Statutory Health Insurance Policy. Jpn. Dent. Sci. Rev..

[50] Allin S., Farmer J., Quiñonez C., Peckham A., Marchildon G., Panteli D., Henschke C., Fattore G., Lamloum D., Holden A.C.L. (2020). Do Health Systems Cover the Mouth? Comparing Dental Care Coverage for Older Adults in Eight Jurisdictions. Health Policy.

[51] Erdsiek F., Waury D., Brzoska P. (2017). Oral Health Behaviour in Migrant and Non-Migrant Adults in Germany: The Utilization of Regular Dental Check-Ups. BMC Oral Health.

[52] Prévention Bucco-Dentaire-La Prévention Dentaire Prend Une Nouvelle Forme Avec «M’T Dents Tous Les Ans!»|Service Public. https://www.service-public.gouv.fr/particuliers/actualites/A17679.

[53] Nay O., Béjean S., Benamouzig D., Bergeron H., Castel P., Ventelou B. (2016). Achieving Universal Health Coverage in France: Policy Reforms and the Challenge of Inequalities. Lancet.

[54] Chevreul K., Berg Brigham K., Durand-Zaleski I., Hernandez-Quevedo C. (2015). France: Health System Review. Health Syst. Transit..

[55] Tubert-Jeannin S., Bénézet L., Mulliez A., Listl S. (2025). The French 100% Santé Reform: Impacts on Dental Care Utilization. J. Dent. Res..

[56] Ardakani M.S.Z., Bayati M. (2025). Global Situation of Oral Health Coverage toward Universal Health Coverage: A Scoping Review. Prev. Med. Rep..

[57] Mazevet M.E., Garyga V., Pitts N.B., Pennington M.W. (2018). The Highly Controversial Payment Reform of Dentists in France: Seeking a New Compromise after the 2017 Strike. Health Policy.

[58] Bas A.C., Azogui-Lévy S. (2019). Evaluation of Children’s Participation in a National Dental Programme in France. Community Dent. Oral Epidemiol..

[59] Bas A.-C. (2023). L’accès aux soins bucco-dentaires dans la réforme 100% santé: Contexte et perspectives. Santé Publique.

[60] Ekici O., Tengilimoglu D., Isik O. (2017). Evaluating the Current Situation of Oral and Dental Healthcare Services in Turkey and Recommending Solutions. Health Policy Technol..

[61] Doğan A., Durukan Köse S. (2025). Oral Health Policy Model for Turkey: How to Deliver Preventive Services?. Front. Health Serv..

[62] (2024). Dental Caries and Associated Factors among Turkish Children and Adults: Findings from the 3rd National Oral Health Survey. Community Dent. Oral Epidemiol..

[63] Çakmakoğlu E.E., Günay A. (2025). Nationwide Prevalence of Dental Caries in Turkish Children: A Meta-Analysis. Children.

[64] Topaloglu-Ak A., Eden E., Frencken J.E. (2009). Managing Dental Caries in Children in Turkey—A Discussion Paper. BMC Oral Health.

[65] FDI World Dental Federation (2019). Thailand Prioritizes Oral Health through Integration into Universal Health Coverage.

[66] Tangcharoensathien V., Witthayapipopsakul W., Panichkriangkrai W., Patcharanarumol W., Mills A. (2018). Health Systems Development in Thailand: A Solid Platform for Successful Implementation of Universal Health Coverage. Lancet.

[67] World Health Organization (2022). Indonesia: Oral Health Country Profile.

[68] World Health Organization (2022). Sri Lanka: Oral Health Country Profile.

[69] World Health Organization (2022). Oral Health Country Profile: Nepal.

[70] Kothia N.R., Bommireddy V.S., Devaki T., Vinnakota N.R., Ravoori S., Sanikommu S., Pachava S. (2015). Assessment of the Status of National Oral Health Policy in India. Int. J. Health Policy Manag..

[71] Rawat R., Aswal G.S., Dwivedi D., Gurumurthy V., Vishwanath S. (2021). Decoding India’s National Oral Health Program-an Appraisal of the Barriers to Quality Dental Care. Int. J. Community Med. Public Health.

[72] Talukdar R., Barman D., Thakkar V., Kanungo S. (2022). Utilization of Dental Care Services among Adult Indian Population: A Meta-Analysis of Evidence from 2011–2022. Health Promot. Perspect..

[73] Okamoto E. (2021). Japan’s Dental Care Facing Population Aging: How Universal Coverage Responds to the Changing Needs of the Elderly. Int. J. Environ. Res. Public Health.

[74] Saito M., Shimazaki Y., Fukai K., Furuta M., Aida J., Ando Y., Miyazaki H., Kambara M. (2020). A Multilevel Analysis of the Importance of Oral Health Instructions for Preventing Tooth Loss: The 8020 Promotion Foundation Study of Japanese Dental Patients. BMC Oral Health.

[75] He C.A., Cheng Y.T., Cheng L., Tao H.U. (2022). Recent Developments and Future Directions of Oral Healthcare System and Dental Public Health System in China in Light of the Current Global Emergency. J. Sichuan Univ. Med. Sci. Ed..

[76] World Health Organization (2022). Cambodia: Oral Health Country Profile.

[77] Organisation for Economic Co-Operation and Development (2022). Health at a Glance: Europe 2022: State of Health in the EU Cycle.

[78] Organisation for Economic Co-Operation and Development (2019). Oral Health and Health Systems: Towards Universal Coverage.

[79] National Health Security Office (2002). National Health Security Act B.E. 2545 (2002).

[80] Ministry of Health (2022). National Oral Health Policy 2022–2030.

[81] Ministry of Health, Labour and Welfare (2021). Overview of the Japanese Health Insurance System.

[82] Federal Office of Public Health (2020). Health Care System in Switzerland.

[83] World Health Organization (2023). World Bank Tracking Universal Health Coverage: 2023 Global Monitoring Report.

[84] Benzian H., Bergman M., Cohen L.K., Hobdell M., Mackay J. (2012). The UN High-Level Meeting on Prevention and Control of Non-Communicable Diseases and Its Significance for Oral Health Worldwide. J. Public Health Dent..

[85] Kassebaum N.J., Bernabé E., Dahiya M., Bhandari B., Murray C.J.L., Marcenes W. (2015). Global Burden of Untreated Caries: A Systematic Review and Metaregression. J. Dent. Res..

[86] Allareddy V., Rampa S., Lee M.K., Allareddy V., Nalliah R.P. (1939). Hospital-Based Emergency Department Visits Involving Dental Conditions: Profile and Predictors of Poor Outcomes and Resource Utilization. J. Am. Dent. Assoc..

[87] Lewis C., Lynch H., Johnston B. (2003). Dental Complaints in Emergency Departments: A National Perspective. Ann. Emerg. Med..

[88] Petersen P.E., Kwan S. (2011). Equity, Social Determinants and Public Health Programmes—The Case of Oral Health. Community Dent. Oral Epidemiol..

[89] Frencken J.E., Sharma P., Stenhouse L., Green D., Laverty D., Dietrich T. (2017). Global Epidemiology of Dental Caries and Severe Periodontitis—A Comprehensive Review. J. Clin. Periodontol..

[90] Baldani M.H., Msc Y.B.E.M., Lawder J.A.d.C., de Lara A.P.I., Rodrigues M.M.A.d.S., Antunes J.L.F. (2011). Inequalities in Dental Services Utilization among Brazilian Low-Income Children: The Role of Individual Determinants. J. Public Health Dent..

[91] Widström E., Eaton K.A. (2004). Oral Healthcare Systems in the Extended European Union. Oral Health Prev. Dent..

[92] Quiñonez C., Grootendorst P. (2011). Equity in Dental Care among Canadian Households. Int. J. Equity Health.

[93] Dehmoobadsharifabadi A., Singhal S., Quiñonez C.R. (2018). Impact of Public Dental Care Spending and Insurance Coverage on Utilization Disparities among Canadian Jurisdictions. J. Public Health Dent..

[94] Stormon N., Do L., Sexton C. (2022). Has the Child Dental Benefits Schedule Improved Access to Dental Care for Australian Children?. Health Soc. Care Community.

[95] Skillman S.M., Doescher M.P., Mouradian W.E., Brunson D.K. (2010). The Challenge to Delivering Oral Health Services in Rural America. J. Public Health Dent..

[96] Nash D.A., Friedman J.W., Kardos T.B., Kardos R.L., Schwarz E., Satur J., Berg D.G., Nasruddin J., Mumghamba E.G., Davenport E.S. (2008). Dental Therapists: A Global Perspective. Int. Dent. J..

[97] Daniel S.J., Kumar S. (2014). Teledentistry: A Key Component in Access to Care. J. Evid.-Based Dent. Pract..

[98] Watt R.G., Daly B., Allison P., Macpherson L.M.D., Venturelli R., Listl S., Weyant R.J., Mathur M.R., Guarnizo-Herreño C.C., Celeste R.K. (2019). Ending the Neglect of Global Oral Health: Time for Radical Action. Lancet.

[99] Petersen P.E., Ogawa H. (2016). Prevention of Dental Caries through the Use of Fluoride—The WHO Approach. Community Dent. Health.

[100] Sheiham A., Watt R.G. (2000). The Common Risk Factor Approach: A Rational Basis for Promoting Oral Health. Community Dent. Oral Epidemiol..

[101] Watt R.G., Sheiham A. (2012). Integrating the Common Risk Factor Approach into a Social Determinants Framework. Community Dent. Oral Epidemiol..

[102] Petersen P.E., Kwan S. (2010). The 7th WHO Global Conference on Health Promotion—Towards Integration of Oral Health (Nairobi, Kenya, 2009). Community Dent. Health.

[103] Kwan S.Y.L., Petersen P.E., Pine C.M., Borutta A. (2005). Health-Promoting Schools: An Opportunity for Oral Health Promotion. Bull. World Health Organ..

[104] Marinho V.C.C., Worthington H.V., Walsh T., Clarkson J.E. (2013). Fluoride Varnishes for Preventing Dental Caries in Children and Adolescents. Cochrane Database Syst. Rev..

[105] Frencken J.E., Peters M.C., Manton D.J., Leal S.C., Gordan V.V., Eden E. (2012). Minimal Intervention Dentistry for Managing Dental Caries—A Review: Report of a FDI Task Group. Int. Dent. J..

[106] Wright J.T., Tampi M.P., Graham L., Estrich C., Crall J.J., Fontana M., Gillette E.J., Nový B.B., Dhar V., Donly K. (2016). Sealants for Preventing and Arresting Pit-and-Fissure Occlusal Caries in Primary and Permanent Molars: A Systematic Review of Randomized Controlled Trials—A Report of the American Dental Association and the American Academy of Pediatric Dentistry. J. Am. Dent. Assoc..

[107] Glick M., Williams D.M., Ben Yahya I. (2021). Vision 2030: Delivering Optimal Oral Health for All.

[108] Eaton K.A., Ramsdale M., Leggett H., Csikar J., Vinall K., Whelton H., Douglas G. (2019). Variations in the Provision and Cost of Oral Healthcare in 11 European Countries: A Case Study. Int. Dent. J..

[109] Ikegami N., Yoo B.-K., Hashimoto H., Matsumoto M., Ogata H., Babazono A., Watanabe R., Shibuya K., Yang B.-M., Reich M.R. (2011). Japanese Universal Health Coverage: Evolution, Achievements, and Challenges. Lancet.

[110] Blümel M., Spranger A., Achstetter K., Maresso A., Busse R. (2020). Germany: Health System Review. Health Syst. Transit..

[111] Borrell L.N., Reynolds J.C., Fleming E., Shah P.D. (2023). Access to Dental Insurance and Oral Health Inequities in the United States. Community Dent. Oral Epidemiol..

[112] Singhal A., Caplan D.J., Jones M.P., Momany E.T., Kuthy R.A., Buresh C.T., Isman R., Damiano P.C. (2015). Eliminating Medicaid Adult Dental Coverage in California Led to Increased Dental Emergency Visits and Associated Costs. Health Aff. Proj. Hope.

[113] Petersen P.E. (2014). Strengthening of Oral Health Systems: Oral Health through Primary Health Care. Med. Princ. Pract..

[114] Dziewulska A., Pawlukowska W., Zawiślak A., Masztalewicz M., Grocholewicz K. (2024). Oral Health in Patients Hospitalized Because of Ischemic Stroke. J. Clin. Med..

[115] Terezakis E., Needleman I., Kumar N., Moles D., Agudo E. (2011). The Impact of Hospitalization on Oral Health: A Systematic Review. J. Clin. Periodontol..

