# Supplementary material for: Structural Embedding of Oral Health Within Pooled Universal Coverage Mechanisms: Where Are We in 2026?
Source: Healthcare (Basel). 2026 Apr 20;14(8):1104. doi: 10.3390/healthcare14081104 (PMC13115761; doi:10.3390/healthcare14081104)
Supplement: Supplementary file 1 [file healthcare-14-01104-s001.zip › Supplementary Table S1. Structural Classification of Oral Health System Integration Across WHO Member States.pdf]

ISO3	Country	WHO_Region	Structural_Category
AFG	Afghanistan	EMRO	Partial or Targeted Integration
ALB	Albania	EURO	Partial or Targeted Integration
DZA	Algeria	AFRO	Predominantly Private / Insurance-Driven
AND	Andorra	EURO	No Data
AGO	Angola	AFRO	Minimal or Emerging Integration
ATG	Antigua and Barbuda	AMRO	Partial or Targeted Integration
ARG	Argentina	AMRO	Partial or Targeted Integration
ARM	Armenia	EURO	No Data
AUS	Australia		Partial or Targeted Integration
AUT	Austria	EURO	Partial or Targeted Integration
AZE	Azerbaijan	EURO	No Data
BHS	Bahamas	AMRO	Partial or Targeted Integration
BHR	Bahrain	EMRO	Partial or Targeted Integration
BGD	Bangladesh	SEARO	Minimal or Emerging Integration
BRB	Barbados	AMRO	Partial or Targeted Integration
BLR	Belarus	EURO	Structural UHC Integration
BEL	Belgium	EURO	Partial or Targeted Integration
BLZ	Belize	AMRO	Partial or Targeted Integration
BEN	Benin	AFRO	Minimal or Emerging Integration
BTN	Bhutan	SEARO	Structural UHC Integration
BOL	Bolivia	AMRO	Partial or Targeted Integration
BIH	Bosnia and Herzegovina	EURO	Partial or Targeted Integration
BWA	Botswana	AFRO	Predominantly Private / Insurance-Driven
BRA	Brazil	AMRO	Structural UHC Integration
BRN	Brunei		Predominantly Private / Insurance-Driven
BGR	Bulgaria	EURO	Predominantly Private / Insurance-Driven
BFA	Burkina Faso	AFRO	No available data
BDI	Burundi	AFRO	Minimal or Emerging Integration
CPV	Cabo Verde	AFRO	Predominantly Private / Insurance-Driven
KHM	Cambodia		Predominantly Private / Insurance-Driven
CMR	Cameroon	AFRO	Minimal or Emerging Integration
CAN	Canada	AMRO	Partial or Targeted Integration
CAF	Central African Republic	AFRO	Minimal or Emerging Integration
TCD	Chad	AFRO	Minimal or Emerging Integration
CHL	Chile	AMRO	Partial or Targeted Integration
CHN	China		Partial or Targeted Integration
COL	Colombia	AMRO	Partial or Targeted Integration
COM	Comoros	AFRO	No available data
COG	Congo	AFRO	No Data
COK	Cook Islands	WPRO	No Data
CRI	Costa Rica	AMRO	Structural UHC Integration
HRV	Croatia	EURO	Partial or Targeted Integration

CUB	Cuba	AMRO	Structural UHC Integration
CYP	Cyprus	EURO	Partial or Targeted Integration
CZE	Czech Republic	EURO	Partial or Targeted Integration
CIV	Côte d’Ivoire	AFRO	Predominantly Private / Insurance-Driven
PRK	Democratic People's Republic of Korea	SEARO	No Data
COD	Democratic Republic of the Congo	AFRO	Minimal or Emerging Integration
DNK	Denmark	EURO	Partial or Targeted Integration
DJI	Djibouti	EMRO	Minimal or Emerging Integration
DMA	Dominica	AMRO	Partial or Targeted Integration
DOM	Dominican Republic	AMRO	Partial or Targeted Integration
ECU	Ecuador	AMRO	Partial or Targeted Integration
EGY	Egypt	EMRO	Partial or Targeted Integration
SLV	El Salvador	AMRO	Partial or Targeted Integration
GNQ	Equatorial Guinea	AFRO	No Data
ERI	Eritrea	AFRO	No available data
EST	Estonia	EURO	Partial or Targeted Integration
SWZ	Eswatini	AFRO	Predominantly Private / Insurance-Driven
ETH	Ethiopia	AFRO	Minimal or Emerging Integration
FJI	Fiji		Predominantly Private / Insurance-Driven
FIN	Finland	EURO	Partial or Targeted Integration
FRA	France	EURO	Partial or Targeted Integration
GAB	Gabon	AFRO	No available data
GMB	Gambia	AFRO	Predominantly Private / Insurance-Driven
GEO	Georgia	EURO	No Data
DEU	Germany	EURO	Structural UHC Integration
GHA	Ghana	AFRO	Predominantly Private / Insurance-Driven
GRC	Greece	EURO	Predominantly Private / Insurance-Driven
GRD	Grenada	AMRO	Partial or Targeted Integration
GTM	Guatemala	AMRO	Minimal or Emerging Integration
GIN	Guinea	AFRO	Minimal or Emerging Integration
GNB	Guinea-Bissau	AFRO	No Data
GUY	Guyana	AMRO	Partial or Targeted Integration
HTI	Haiti	AMRO	Minimal or Emerging Integration
HND	Honduras	AMRO	Partial or Targeted Integration
HUN	Hungary	EURO	Partial or Targeted Integration
ISL	Iceland	EURO	Predominantly Private / Insurance-Driven
IND	India	SEARO	Partial or Targeted Integration
IDN	Indonesia	SEARO	Partial or Targeted Integration
IRN	Iran		Partial or Targeted Integration
IRQ	Iraq	EMRO	Partial or Targeted Integration
IRL	Ireland	EURO	Partial or Targeted Integration

ISR	Israel	EURO	Partial or Targeted Integration
ITA	Italy	EURO	Partial or Targeted Integration
JAM	Jamaica	AMRO	Partial or Targeted Integration
JPN	Japan		Structural UHC Integration
JOR	Jordan	EMRO	Partial or Targeted Integration
KAZ	Kazakhstan	EURO	Partial or Targeted Integration
KEN	Kenya	AFRO	Predominantly Private / Insurance-Driven
KIR	Kiribati		No Data
KWT	Kuwait	EMRO	Partial or Targeted Integration
KGZ	Kyrgyzstan	EURO	Minimal or Emerging Integration
LAO	Lao PDR		Predominantly Private / Insurance-Driven
LVA	Latvia	EURO	Partial or Targeted Integration
LBN	Lebanon	EMRO	Minimal or Emerging Integration
LSO	Lesotho	AFRO	Predominantly Private / Insurance-Driven
LBR	Liberia	AFRO	No available data
LBY	Libya	EMRO	No Data
LTU	Lithuania	EURO	Partial or Targeted Integration
LUX	Luxembourg	EURO	Partial or Targeted Integration
MDG	Madagascar	AFRO	Minimal or Emerging Integration
MWI	Malawi	AFRO	Predominantly Private / Insurance-Driven
MYS	Malaysia		Partial or Targeted Integration
MDV	Maldives	SEARO	Partial or Targeted Integration
MLI	Mali	AFRO	No available data
MLT	Malta	EURO	Predominantly Private / Insurance-Driven
MHL	Marshall Islands		No Data
MRT	Mauritania	AFRO	Minimal or Emerging Integration
MUS	Mauritius	AFRO	Structural UHC Integration
MEX	Mexico	AMRO	Partial or Targeted Integration
FSM	Micronesia		No available data
MDA	Moldova	EURO	No Data
MCO	Monaco	EURO	No Data
MNG	Mongolia		Partial or Targeted Integration
MNE	Montenegro	EURO	Partial or Targeted Integration
MAR	Morocco	EMRO	Partial or Targeted Integration
MOZ	Mozambique	AFRO	Minimal or Emerging Integration
MMR	Myanmar	SEARO	Minimal or Emerging Integration
NAM	Namibia	AFRO	Predominantly Private / Insurance-Driven
NRU	Nauru		No Data
NPL	Nepal	SEARO	Minimal or Emerging Integration
NLD	Netherlands	EURO	Predominantly Private / Insurance-Driven
NZL	New Zealand		Partial or Targeted Integration
NIC	Nicaragua	AMRO	Structural UHC Integration
NER	Niger	AFRO	No Data

NGA	Nigeria	AFRO	Minimal or Emerging Integration
NIU	Niue	WPRO	No Data
MKD	North Macedonia	EURO	Structural UHC Integration
NOR	Norway	EURO	Partial or Targeted Integration
OMN	Oman	EMRO	Partial or Targeted Integration
PAK	Pakistan	EMRO	Partial or Targeted Integration
PLW	Palau		No Data
PAN	Panama	AMRO	Partial or Targeted Integration
PNG	Papua New Guinea		No available data
PRY	Paraguay	AMRO	Partial or Targeted Integration
PER	Peru	AMRO	Partial or Targeted Integration
PHL	Philippines		Partial or Targeted Integration
POL	Poland	EURO	Partial or Targeted Integration
PRT	Portugal	EURO	Partial or Targeted Integration
QAT	Qatar	EMRO	Partial or Targeted Integration
ROU	Romania	EURO	Partial or Targeted Integration
RUS	Russia	EURO	No Data
RWA	Rwanda	AFRO	Predominantly Private / Insurance-Driven
KNA	Saint Kitts and Nevis	AMRO	Partial or Targeted Integration
LCA	Saint Lucia	AMRO	Partial or Targeted Integration
VCT	Saint Vincent and the Grenadines	AMRO	Partial or Targeted Integration
WSM	Samoa		Predominantly Private / Insurance-Driven
SMR	San Marino	EURO	No Data
STP	Sao Tome and Principe	AFRO	No Data
SAU	Saudi Arabia	EMRO	Predominantly Private / Insurance-Driven
SEN	Senegal	AFRO	Predominantly Private / Insurance-Driven
SRB	Serbia	EURO	Partial or Targeted Integration
SYC	Seychelles	AFRO	Structural UHC Integration
SLE	Sierra Leone	AFRO	No available data
SGP	Singapore		Structural UHC Integration
SVK	Slovakia	EURO	Predominantly Private / Insurance-Driven
SVN	Slovenia	EURO	Partial or Targeted Integration
SLB	Solomon Islands		No available data
SOM	Somalia	EMRO	Minimal or Emerging Integration
ZAF	South Africa	AFRO	Partial or Targeted Integration
KOR	South Korea		Structural UHC Integration
SSD	South Sudan	AFRO	Minimal or Emerging Integration
ESP	Spain	EURO	Partial or Targeted Integration
LKA	Sri Lanka	SEARO	Structural UHC Integration
SDN	Sudan	EMRO	Minimal or Emerging Integration
SUR	Suriname	AMRO	Partial or Targeted Integration
SWE	Sweden	EURO	Partial or Targeted Integration

CHE	Switzerland	EURO	Predominantly Private / Insurance-Driven
SYR	Syrian Arab Republic	EMRO	Partial or Targeted Integration
TJK	Tajikistan	EURO	Partial or Targeted Integration
TZA	Tanzania	AFRO	Predominantly Private / Insurance-Driven
THA	Thailand	SEARO	Partial or Targeted Integration
TLS	Timor-Leste	SEARO	Minimal or Emerging Integration
TGO	Togo	AFRO	Predominantly Private / Insurance-Driven
TON	Tonga		Predominantly Private / Insurance-Driven
TTO	Trinidad and Tobago	AMRO	Partial or Targeted Integration
TUN	Tunisia	EMRO	Structural UHC Integration
TUR	Turkey	EURO	Predominantly Private / Insurance-Driven
TKM	Turkmenistan	EURO	Minimal or Emerging Integration
TUV	Tuvalu		No available data
UGA	Uganda	AFRO	Predominantly Private / Insurance-Driven
UKR	Ukraine	EURO	Partial or Targeted Integration
ARE	United Arab Emirates	EMRO	Predominantly Private / Insurance-Driven
GBR	United Kingdom	EURO	Partial or Targeted Integration
USA	United States	AMRO	Predominantly Private / Insurance-Driven
URY	Uruguay	AMRO	Partial or Targeted Integration
UZB	Uzbekistan	EURO	Minimal or Emerging Integration
VUT	Vanuatu		No available data
VEN	Venezuela	AMRO	Partial or Targeted Integration
VNM	Vietnam	WPRO	Partial or Targeted Integration
YEM	Yemen	EMRO	Partial or Targeted Integration
ZMB	Zambia	AFRO	Predominantly Private / Insurance-Driven
ZWE	Zimbabwe	AFRO	Predominantly Private / Insurance-Driven

Key Source Reference:

https://cdn.who.int/media/docs/default-source/country-profiles/oral-health/oral-health-afg-2022-country-profile.pdf?download=true&sfvrsn=f8c624e6_6

https://cdn.who.int/media/docs/default-source/country-profiles/oral-health/oral-health-alb-2022-country-profile.pdf?download=true&sfvrsn=43fcba5b_4

https://cdn.who.int/media/docs/default-source/country-profiles/oral-health/oral-health-and-2022-country-profile.pdf

https://cdn.who.int/media/docs/default-source/country-profiles/oral-health/oral-health-atg-2022-country-profile.pdf?download=true&sfvrsn=fe2fc294_6

https://cdn.who.int/media/docs/default-source/country-profiles/oral-health/oral-health-arg-2022-country-profile.pdf

https://cdn.who.int/media/docs/default-source/country-profiles/oral-health/oral-health-arm-2022-country-profile.pdf

https://cdn.who.int/media/docs/default-source/country-profiles/oral-health/oral-health-aut-2022-country-profile.pdf

https://cdn.who.int/media/docs/default-source/country-profiles/oral-health/oral-health-aze-2022-country-profile.pdf

https://cdn.who.int/media/docs/default-source/country-profiles/oral-health/oral-health-bhs-2022-country-profile.pdf

https://cdn.who.int/media/docs/default-source/country-profiles/oral-health/oral-health-bhr-2022-country-profile.pdf?sfvrsn=a445b75c_6

https://cdn.who.int/media/docs/default-source/country-profiles/oral-health/oral-health-bgd-2022-country-profile.pdf?download=true&sfvrsn=48e7b86c_6

https://cdn.who.int/media/docs/default-source/country-profiles/oral-health/oral-health-brb-2022-country-profile.pdf

https://cdn.who.int/media/docs/default-source/country-profiles/oral-health/oral-health-blr-2022-country-profile.pdf?download=true&sfvrsn=272423e2_6

https://cdn.who.int/media/docs/default-source/country-profiles/oral-health/oral-health-bel-2022-country-profile.pdf

https://cdn.who.int/media/docs/default-source/country-profiles/oral-health/oral-health-blz-2022-country-profile.pdf?download=true&sfvrsn=65ef0bc1_4

https://cdn.who.int/media/docs/default-source/country-profiles/oral-health/oral-health-btn-2022-country-profile.pdf?download=true&sfvrsn=9a20a767_6

https://cdn.who.int/media/docs/default-source/country-profiles/oral-health/oral-health-bol-2022-country-profile.pdf?download=true&sfvrsn=84390ef1_4

https://cdn.who.int/media/docs/default-source/country-profiles/oral-health/oral-health-bih-2022-country-profile.pdf?sfvrsn=7208025b_6

https://cdn.who.int/media/docs/default-source/country-profiles/oral-health/oral-health-bra-2022-country-profile.pdf

https://cdn.who.int/media/docs/default-source/country-profiles/oral-health/oral-health-bgr-2022-country-profile.pdf

https://cdn.who.int/media/docs/default-source/country-profiles/oral-health/oral-health-can-2022-country-profile.pdf

https://cdn.who.int/media/docs/default-source/country-profiles/oral-health/oral-health-chl-2022-country-profile.pdf

https://cdn.who.int/media/docs/default-source/country-profiles/oral-health/oral-health-col-2022-country-profile.pdf

https://cdn.who.int/media/docs/default-source/country-profiles/oral-health/oral-health-cri-2022-country-profile.pdf?download=true&sfvrsn=f31860f9_7

https://cdn.who.int/media/docs/default-source/country-profiles/oral-health/oral-health-hrv-2022-country-profile.pdf

https://cdn.who.int/media/docs/default-source/country-profiles/oral-health/oral-health-cub-2022-country-profile.pdf?sfvrsn=1682ae8f_9

https://cdn.who.int/media/docs/default-source/country-profiles/oral-health/oral-health-cyp-2022-country-profile.pdf

https://cdn.who.int/media/docs/default-source/country-profiles/oral-health/oral-health-cze-2022-country-profile.pdf

https://cdn.who.int/media/docs/default-source/country-profiles/oral-health/oral-health-democratic-peoples-republic-of-korea-2022.pdf?download=true&sfvrsn=803dd41d_9

https://cdn.who.int/media/docs/default-source/country-profiles/oral-health/oral-health-dnk-2022-country-profile.pdf

https://cdn.who.int/media/docs/default-source/country-profiles/oral-health/oral-health-dji-2022-country-profile.pdf?download=true&sfvrsn=efe1aa60_6

https://cdn.who.int/media/docs/default-source/country-profiles/oral-health/oral-health-dma-2022-country-profile.pdf?download=true&sfvrsn=2c4ba198_5

https://cdn.who.int/media/docs/default-source/country-profiles/oral-health/oral-health-dom-2022-country-profile.pdf?download=true&sfvrsn=e20b3320_7

https://cdn.who.int/media/docs/default-source/country-profiles/oral-health/oral-health-ecu-2022-country-profile.pdf?download=true&sfvrsn=8085cec4_7

https://cdn.who.int/media/docs/default-source/country-profiles/oral-health/oral-health-egy-2022-country-profile.pdf

https://cdn.who.int/media/docs/default-source/country-profiles/oral-health/oral-health-slv-2022-country-profile.pdf?download=true&sfvrsn=9c3b8d3d_7

https://cdn.who.int/media/docs/default-source/country-profiles/oral-health/oral-health-est-2022-country-profile.pdf

https://cdn.who.int/media/docs/default-source/country-profiles/oral-health/oral-health-fin-2022-country-profile.pdf

https://cdn.who.int/media/docs/default-source/country-profiles/oral-health/oral-health-fra-2022-country-profile.pdf

https://cdn.who.int/media/docs/default-source/country-profiles/oral-health/oral-health-geo-2022-country-profile.pdf

https://cdn.who.int/media/docs/default-source/country-profiles/oral-health/oral-health-deu-2022-country-profile.pdf

https://cdn.who.int/media/docs/default-source/country-profiles/oral-health/oral-health-grc-2022-country-profile.pdf

https://cdn.who.int/media/docs/default-source/country-profiles/oral-health/oral-health-grd-2022-country-profile.pdf?sfvrsn=4cf7aa4e_9

https://cdn.who.int/media/docs/default-source/country-profiles/oral-health/oral-health-gtm-2022-country-profile.pdf?download=true&sfvrsn=84ac3b75_7

https://cdn.who.int/media/docs/default-source/country-profiles/oral-health/oral-health-guy-2022-country-profile.pdf?download=true&sfvrsn=e14f9f22_8

https://cdn.who.int/media/docs/default-source/country-profiles/oral-health/oral-health-hti-2022-country-profile.pdf?download=true&sfvrsn=8edb92b5_7

https://cdn.who.int/media/docs/default-source/country-profiles/oral-health/oral-health-hnd-2022-country-profile.pdf?download=true&sfvrsn=4cc26ca0_8

https://cdn.who.int/media/docs/default-source/country-profiles/oral-health/oral-health-hun-2022-country-profile.pdf

https://cdn.who.int/media/docs/default-source/country-profiles/oral-health/oral-health-isl-2022-country-profile.pdf

https://cdn.who.int/media/docs/default-source/country-profiles/oral-health/oral-health-ind-2022-country-profile.pdf

https://cdn.who.int/media/docs/default-source/country-profiles/oral-health/oral-health-idn-2022-country-profile.pdf

https://cdn.who.int/media/docs/default-source/country-profiles/oral-health/oral-health-irn-2022-country-profile.pdf

https://cdn.who.int/media/docs/default-source/country-profiles/oral-health/oral-health-irq-2022-country-profile.pdf?sfvrsn=64da2bc3_10

https://cdn.who.int/media/docs/default-source/country-profiles/oral-health/oral-health-irl-2022-country-profile.pdf

https://cdn.who.int/media/docs/default-source/country-profiles/oral-health/oral-health-ita-2022-country-profile.pdf

https://cdn.who.int/media/docs/default-source/country-profiles/oral-health/oral-health-jam-2022-country-profile.pdf?download=true&sfvrsn=9d30a7ed_7

https://cdn.who.int/media/docs/default-source/country-profiles/oral-health/oral-health-jor-2022-country-profile.pdf?download=true&sfvrsn=9d29213_7

https://cdn.who.int/media/docs/default-source/country-profiles/oral-health/oral-health-kwt-2022-country-profile.pdf

https://cdn.who.int/media/docs/default-source/country-profiles/oral-health/oral-health-lva-2022-country-profile.pdf

https://cdn.who.int/media/docs/default-source/country-profiles/oral-health/oral-health-lbn-2022-country-profile.pdf

https://cdn.who.int/media/docs/default-source/country-profiles/oral-health/oral-health-lby-2022-country-profile.pdf?download=true&sfvrsn=fec75e8d_6

https://cdn.who.int/media/docs/default-source/country-profiles/oral-health/oral-health-ltu-2022-country-profile.pdf

https://cdn.who.int/media/docs/default-source/country-profiles/oral-health/oral-health-lux-2022-country-profile.pdf

https://cdn.who.int/media/docs/default-source/country-profiles/oral-health/oral-health-mdv-2022-country-profile.pdf?download=true&sfvrsn=c12544a0_4

https://cdn.who.int/media/docs/default-source/country-profiles/oral-health/oral-health-mlt-2022-country-profile.pdf

https://cdn.who.int/media/docs/default-source/country-profiles/oral-health/oral-health-mex-2022-country-profile.pdf

https://cdn.who.int/media/docs/default-source/country-profiles/oral-health/oral-health-mda-2022-country-profile.pdf

https://cdn.who.int/media/docs/default-source/country-profiles/oral-health/oral-health-mco-2022-country-profile.pdf

https://cdn.who.int/media/docs/default-source/country-profiles/oral-health/oral-health-mne-2022-country-profile.pdf?download=true&sfvrsn=97131023_6

https://cdn.who.int/media/docs/default-source/country-profiles/oral-health/oral-health-mar-2022-country-profile.pdf?download=true&sfvrsn=ef733e2e_4

https://cdn.who.int/media/docs/default-source/country-profiles/oral-health/oral-health-mmr-2022-country-profile.pdf?download=true&sfvrsn=64b185fd_6

https://cdn.who.int/media/docs/default-source/country-profiles/oral-health/oral-health-npl-2022-country-profile.pdf?download=true&sfvrsn=3038153_6

https://cdn.who.int/media/docs/default-source/country-profiles/oral-health/oral-health-nld-2022-country-profile.pdf

https://cdn.who.int/media/docs/default-source/country-profiles/oral-health/oral-health-nic-2022-country-profile.pdf?download=true&sfvrsn=5ebdf9ad_9

https://cdn.who.int/media/docs/default-source/country-profiles/oral-health/oral-health-mkd-2022-country-profile.pdf?sfvrsn=c2828b74_9

https://cdn.who.int/media/docs/default-source/country-profiles/oral-health/oral-health-nor-2022-country-profile.pdf

https://cdn.who.int/media/docs/default-source/country-profiles/oral-health/oral-health-omn-2022-country-profile.pdf

https://cdn.who.int/media/docs/default-source/country-profiles/oral-health/oral-health-pak-2022-country-profile.pdf

https://cdn.who.int/media/docs/default-source/country-profiles/oral-health/oral-health-pan-2022-country-profile.pdf?download=true&sfvrsn=628fb9b3_9

https://cdn.who.int/media/docs/default-source/country-profiles/oral-health/oral-health-pry-2022-country-profile.pdf?sfvrsn=b740b49d_9

https://cdn.who.int/media/docs/default-source/country-profiles/oral-health/oral-health-per-2022-country-profile.pdf

https://cdn.who.int/media/docs/default-source/country-profiles/oral-health/oral-health-pol-2022-country-profile.pdf

https://cdn.who.int/media/docs/default-source/country-profiles/oral-health/oral-health-prt-2022-country-profile.pdf

https://cdn.who.int/media/docs/default-source/country-profiles/oral-health/oral-health-qat-2022-country-profile.pdf

https://cdn.who.int/media/docs/default-source/country-profiles/oral-health/oral-health-rou-2022-country-profile.pdf

https://cdn.who.int/media/docs/default-source/country-profiles/oral-health/oral-health-kna-2022-country-profile.pdf?download=true&sfvrsn=9297af0_7

https://cdn.who.int/media/docs/default-source/country-profiles/oral-health/oral-health-lca-2022-country-profile.pdf?download=true&sfvrsn=1d91f5f0_7

https://cdn.who.int/media/docs/default-source/country-profiles/oral-health/oral-health-vct-2022-country-profile.pdf?download=true&sfvrsn=89d3578d_7

https://cdn.who.int/media/docs/default-source/country-profiles/oral-health/oral-health-smr-2022-country-profile.pdf

https://cdn.who.int/media/docs/default-source/country-profiles/oral-health/oral-health-sau-2022-country-profile.pdf

https://cdn.who.int/media/docs/default-source/country-profiles/oral-health/oral-health-srb-2022-country-profile.pdf?download=true&sfvrsn=afb21392_5

https://cdn.who.int/media/docs/default-source/country-profiles/oral-health/oral-health-svk-2022-country-profile.pdf

https://cdn.who.int/media/docs/default-source/country-profiles/oral-health/oral-health-svn-2022-country-profile.pdf

https://cdn.who.int/media/docs/default-source/country-profiles/oral-health/oral-health-som-2022-country-profile.pdf?download=true&sfvrsn=c2bdd2b5_5

https://cdn.who.int/media/docs/default-source/country-profiles/oral-health/oral-health-esp-2022-country-profile.pdf

https://cdn.who.int/media/docs/default-source/country-profiles/oral-health/oral-health-lka-2022-country-profile.pdf

https://cdn.who.int/media/docs/default-source/country-profiles/oral-health/oral-health-sdn-2022-country-profile.pdf?download=true&sfvrsn=206e5160_7

https://cdn.who.int/media/docs/default-source/country-profiles/oral-health/oral-health-sur-2022-country-profile.pdf?download=true&sfvrsn=1c73834c_7

https://cdn.who.int/media/docs/default-source/country-profiles/oral-health/oral-health-swe-2022-country-profile.pdf

https://cdn.who.int/media/docs/default-source/country-profiles/oral-health/oral-health-che-2022-country-profile.pdf

https://cdn.who.int/media/docs/default-source/country-profiles/oral-health/oral-health-syr-2022-country-profile.pdf?download=true&sfvrsn=41852bdc_7

https://cdn.who.int/media/docs/default-source/country-profiles/oral-health/oral-health-tha-2022-country-profile.pdf?download=true&sfvrsn=e72cab7d_7

https://cdn.who.int/media/docs/default-source/country-profiles/oral-health/oral-health-tls-2022-country-profile.pdf?download=true&sfvrsn=a8c0e9b3_6

https://cdn.who.int/media/docs/default-source/country-profiles/oral-health/oral-health-tto-2022-country-profile.pdf?sfvrsn=f860371a_7

https://cdn.who.int/media/docs/default-source/country-profiles/oral-health/oral-health-tun-2022-country-profile.pdf?download=true&sfvrsn=2d019040_5

https://cdn.who.int/media/docs/default-source/country-profiles/oral-health/oral-health-ukr-2022-country-profile.pdf?do=&sfvrsn=275bef62_5

https://cdn.who.int/media/docs/default-source/country-profiles/oral-health/oral-health-are-2022-country-profile.pdf

https://cdn.who.int/media/docs/default-source/country-profiles/oral-health/oral-health-gbr-2022-country-profile.pdf

https://cdn.who.int/media/docs/default-source/country-profiles/oral-health/oral-health-usa-2022-country-profile.pdf

https://cdn.who.int/media/docs/default-source/country-profiles/oral-health/oral-health-ury-2022-country-profile.pdf?download=true&sfvrsn=3e461eef_5

https://cdn.who.int/media/docs/default-source/country-profiles/oral-health/oral-health-ven-2022-country-profile.pdf?download=true&sfvrsn=896a7814_10

https://cdn.who.int/media/docs/default-source/country-profiles/oral-health/oral-health-yem-2022-country-profile.pdf?sfvrsn=e589a5d2_12
