# Supplementary material for: Structural Embedding of Oral Health Within Pooled Universal Coverage Mechanisms: Where Are We in 2026?
Source: Healthcare (Basel). 2026 Apr 20;14(8):1104. doi: 10.3390/healthcare14081104 (PMC13115761; doi:10.3390/healthcare14081104)
Supplement: Supplementary file 1 [file healthcare-14-01104-s001.zip › Supplementary Table S2. Regional Distribution of Structural Integration Models.pdf]

WHO_Region	AFRO	AMRO	EMRO	EURO	SEARO	WPRO	**Worldwide**
Structural UHC Integration	8%	11%	5%	6%	18%	21%	**10%**
Partial or Targeted Integration	10%	77%	59%	55%	36.5%	29%	**44%**
Predominantly Private / Insurance-Driven	38%	3%	9%	14%	0%	21%	**17%**
Minimal or Emerging Integration	31%	9%	18%	5%	36.5%	4%	**15%**
No Data	13%	0%	9%	20%	9%	25%	**14%**
