# Supplementary material for: Structural Embedding of Oral Health Within Pooled Universal Coverage Mechanisms: Where Are We in 2026?
Source: Healthcare (Basel). 2026 Apr 20;14(8):1104. doi: 10.3390/healthcare14081104 (PMC13115761; doi:10.3390/healthcare14081104)
Supplement: Supplementary file 1 [file healthcare-14-01104-s001.zip › Supplementary Table S3. Comparison of national oral health policies and implementation across selected countries.pdf]

Supplementary Table S3. Comparison of national oral health policies and implementation across selected countries

AFRO – African Region						
**Income level**	**Country**	**National Oral Health Policy / Framework**	**Integration into Health System**	**Key Preventive Initiatives**	**Financing, Workforce & Surveillance**	**Notable Targets, Outcomes, or Gaps**
**High**	Seychelles [1]	National health policy includes oral health	Comprehensive public dental services	Preventive programs	Tax-funded; small but adequate workforce	Small-state exemplar
**Upper-Middle**	South Africa [2,3]	National oral health policy	Broad service inclusion in public sector	Fluoridation; school programs	Mixed financing; surveillance present	Persistent inequalities
**Lower-Middle**	Kenya [4,5]	National Oral Health Policy 2022–2030, aligned with WHO Global Strategy.	Oral health gradually being integrated into primary care within UHC pilot schemes; services still largely hospital- or private-based.	Expanding school-based oral health education; community outreach for oral cancer awareness; consideration of population-level fluoridation strategies.	Mixed financing with substantial out-of-pocket payment; significant workforce shortages; limited but developing surveillance capacity.	Policy targets include reductions in dental caries and periodontal disease by 2030; implementation constrained by infrastructure and workforce limitations.
**Low**	Uganda [6,7]	National oral health strategy	Preventive services in public facilities	Community education	High OOP; severe workforce shortages; weak surveillance	Limited coverage

AMRO – Region of the Americas						
**Income level**	**Country**	**National Oral Health Policy / Framework**	**Integration into Health System**	**Key Preventive Initiatives**	**Financing, Workforce & Surveillance**	**Notable Targets, Outcomes, or Gaps**
**High**	United States [8–13]	No single comprehensive national oral health policy; oral health addressed through federal reports and state-level initiatives.	No universal dental coverage; dental care largely dependent on private insurance or out-of-pocket payment; public coverage mainly for children (Medicaid/CHIP).	Community water fluoridation (~73% of population on public water systems); national health promotion campaigns on oral hygiene.	Fragmented financing with high out-of-pocket costs; large private workforce but uneven access; surveillance through national surveys (NHANES).	Persistent disparities by income, race, and insurance status; higher prevalence of untreated disease and delayed care compared with UHC systems.
	Canada [14–16]	Historically fragmented; emerging national framework following federal dental care reforms	Preventive and essential dental services expanding under publicly funded Canadian Dental Care Plan	Community water fluoridation; population-level prevention through primary care	Mixed public/private financing; strong workforce; historically limited national surveillance	Major post-2023 policy shift toward universal coverage; implementation and equity impacts under evaluation. Full dental and medical coverage (including travel) for some First Nations communities in remote Québec; adult public coverage expanding nationally.

**Upper-Middle**	Mexico [17]	National oral health strategies integrated within broader health policy	Preventive, essential, and advanced dental services included for priority groups	Community fluoridation; school oral health programs	Public insurance plus OOP payments; workforce adequate; surveillance variable	Coverage remains unequal despite formal inclusion
	Brazil [18–20]	National Oral Health Policy (Brasil Sorridente), launched in 2004.	Oral health fully embedded within the Family Health Strategy, with dedicated oral health teams in primary care nationwide.	Community water fluoridation in many cities; fluoride varnish programs; large-scale school- and community-based oral health education.	Tax-funded public system; strong reliance on primary care dentists and auxiliaries; national surveillance systems support monitoring, though implementation varies regionally.	Marked expansion of access and improvements in child oral health; reductions in edentulism, but regional inequalities and recent fiscal constraints challenge long-term sustainability.
**Lower-Middle**	Haiti [21]	No comprehensive national oral health policy	Minimal integration; services largely emergency-based	Limited preventive initiatives	Predominantly out-of-pocket; severe workforce shortages; no routine surveillance	Very limited access; major data gaps

**EMRO – Eastern Mediterranean Region**						
**Income level**	**Country**	**National Oral Health Policy / Framework**	**Integration into Health System**	**Key Preventive Initiatives**	**Financing, Workforce & Surveillance**	**Notable Targets, Outcomes, or Gaps**
**High**	United Arab Emira	No unified national oral health policy; oral	Services mainly through public	School-based and child-focused programs	Predominantly private financing with public coverage for	High prevalence of dental caries and

	tes [22–24]	health addressed within federal wellbeing and NCD strategies, with implementation largely at emirate level	authority clinics and strong private sector; preventive care available but not consistently embedded in UHC-type packages.	(varnish, education) in some emirates; sugar-sweetened beverage tax and lifestyle prevention measures under national NCD and wellbeing strategies.	citizens; Good urban service availability, but preventive service utilization remains suboptimal; surveillance largely based on isolated emirate-level or academic studies; no unified national oral health monitoring system.	ECC; risk factors include frequent snacking, lower maternal education, low SES and poor brushing; lack of coordinated national prevention policy.
	Saudi Arabia [25]	No single stand-alone national oral health policy; oral health priorities embedded in broader health and Saudi Vision 2030 planning.	Large public network plus extensive private sector; dental care mainly treatment-oriented, with emerging pilots integrating prevention into pediatric and primary care (e.g., COHI).	Sugar-sweetened beverage tax; hospital-based Child Oral Health Initiative with electronic referrals and fluoride varnish; small-scale health-promotion and school activities.	Mixed public funding and private fees; workforce shortages and urban–rural maldistribution; oral health promotion under-resourced; surveillance fragmented and largely research-driven.	Very high levels of caries despite high income; inequities by region and SES; limited population-wide preventive programs and weak integration of promotion into primary care.
**Upper-Middle**	Libya [26,27]	No clear national oral health policy; oral health notionally within primary health care but without defined	Oral health "integrated" administratively into medical services, but most care is treatment-based in private clinics; public sector focuses on	No structured national prevention; very limited organized school or community programs.	Public services government-funded but poorly organized; private sector self-funded and dominant; severe shortage of hygienists/technicians; virtually	Very high caries burden (≈70% of preschool children); large unmet need; urgent call for policies and

		target groups.	emergencies and extractions.		no routine surveillance.	system reform.

**EURO – European Region**						
**Income level**	**Country**	**National Oral Health Policy / Framework**	**Integration into Health System**	**Key Preventive Initiatives**	**Financing, Workforce & Surveillance**	**Notable Targets, Outcomes, or Gaps**
**High**	United Kingdom [28–31]	Oral health integrated into NHS dentistry; national clinical guidance (Delivering Better Oral Health toolkit).	Universal coverage for basic dental services via the National Health Service (NHS); preventive care formally emphasized, with co-payments for most adults.	Widespread use of fluoride toothpaste; longstanding school dental programs; sugar-sweetened beverage levy (introduced in 2018).	Publicly funded system with mixed incentives; workforce shortages and uneven geographic distribution constrain access; national surveillance through periodic child dental health surveys.	Historically low average levels of childhood caries, but persistent socioeconomic gradients remain; ongoing NHS dental contract reforms aim to strengthen prevention and improve access.
	Switzerland [32,33]	No unified national oral health policy; oral health responsibilities largely devolved to cantons.	Dental care excluded from mandatory health insurance; services primarily delivered through private practices; limited public coverage for children and	School-based dental services and prevention programs in several cantons; strong emphasis on individual oral hygiene and	Predominantly out-of-pocket financing; strong private dental workforce; national surveillance limited, with data mainly from periodic surveys and	Overall good oral health outcomes, but persistent socioeconomic inequalities and access barriers related to cost; prevention and coverage vary substantially across cantons.

			vulnerable groups at cantonal level.	fluoride toothpaste use.	cantonal sources.	
	Germany [30,34–37]	No single stand-alone oral health policy; oral health governed through statutory health insurance framework	Comprehensive dental care included in statutory health insurance with regulated benefits and co-payments	Preventive check-ups, fluoride toothpaste use, school dental services	Social insurance financing; strong private workforce; robust routine data via insurance claims	High overall coverage and outcomes; absence of unified national oral health strategy; persistent social gradients
	France [38–45]	Historically no single comprehensive oral health policy, with recent reforms partially compensating for the absence of a unified national strategy; reliance on insurance mechanisms and targeted programs.	Dental care included in national health insurance, traditionally with substantial co-payments; recent reforms expanded coverage.	M'T Dents preventive program (expanded to annual visits ages 3–24); school-based education; strong tobacco control to reduce oral cancer.	Social insurance financing; predominantly private dental workforce; limited national oral health surveillance; governance fragmented across insurance, public health, and professional bodies.	100% Santé reform eliminated out-of-pocket costs for selected prosthetic care, increasing utilization; absence of a unified national strategy has contributed to fragmented implementation.
**Upper-Middle**	Türkiye [46–50]	Partial national oral health framework	Preventive and essential services included in public	School-based education; basic	Mixed public/private financing; workforce unevenly	Partial integration; regional inequalities; limited

			system, with access limitations	preventive services	distributed; limited surveillance	preventive reach

**SEARO – South-East Asia Region**						
**Income level**	**Country**	**National Oral Health Policy / Framework**	**Integration into Health System**	**Key Preventive Initiatives**	**Financing, Workforce & Surveillance**	**Notable Targets, Outcomes, or Gaps**
**Upper-Middle**	Thailand [51,52]	National oral health policies aligned with universal health coverage since early 2000s.	Universal Coverage Scheme (UCS) includes preventive and basic restorative dental services as part of the benefits package.	School-based fluoride rinse and sealant programs; routine dental check-ups; community oral health promotion.	Predominantly tax-financed; strong primary care dental workforce, though rural distribution remains uneven; routine national monitoring of service utilization and oral health indicators.	High utilization of dental services and comparatively low levels of untreated decay among children; ongoing workforce training to address rural access gaps.
	Indonesia [53]	National oral health strategies	Partial integration within public insurance	Community promotion; school programs	Mixed financing; workforce shortages; limited surveillance	Coverage uneven across regions
**Lower-Middle**	Sri Lanka [54]	National oral health policy aligned with UHC	Preventive, essential, and advanced dental care integrated into public	School dental services; routine check-ups	Tax-funded; strong PHC orientation; national monitoring	Strong outcomes; relatively equitable access

			health system			
	Nepal [55]	Partial oral health framework	Preventive services in public sector; no UHC dental package	School education; limited fluoride exposure	High OOP; severe workforce shortages; weak surveillance	Very high unmet need; limited policy implementation
	India [56–58]	National Oral Health Program recognized within national health policy, with periodic updates.	Not fully integrated into UHC; public dental services exist but reach is limited; majority of care delivered privately.	State-level school dental programs; national tobacco control initiatives; pilot preventive interventions in high-risk groups.	Predominantly out-of-pocket financing; very large but unevenly distributed dental workforce; limited routine surveillance at national level.	Very high burden of dental caries and oral cancer; ongoing efforts focus on expanding rural services and integrating basic oral health into primary health screenings.

**WPRO – Western Pacific Region**						
**Income level**	**Country**	**National Oral Health Policy / Framework**	**Integration into Health System**	**Key Preventive Initiatives**	**Financing, Workforce & Surveillance**	**Notable Targets, Outcomes, or Gaps**
**High**	Japan [59,60]	National oral health policy (e.g. 8020 strategy)	Comprehensive dental care included in universal health insurance	Lifecourse prevention; school dental programs	Social insurance financing; strong surveillance	Excellent outcomes; ageing-related challenges
**Upper-Middle**	China [6,42,61]	National oral health plans	Preventive and essential services included variably	School education; pilot community programs	Mixed financing; large workforce; uneven data	Large urban–rural disparities

**Lower-Middle**	Cambodia [6,62]	National oral health plan	Preventive services only in public sector	School-based education	High OOP; limited workforce; minimal surveillance	Major access gaps

1. World Health Organization *Seychelles: Oral Health Country Profile*; World Health Organization: Geneva, Switzerland, 2022;
2. World Health Organization *South Africa: Oral Health Country Profile*; World Health Organization: Geneva, Switzerland, 2022;
3. van Wyk, P.J.; van Wyk, C. Oral Health in South Africa. *Int. Dent. J.* **2004**, *54*, 373–377, doi:10.1111/j.1875-595x.2004.tb00014.x.
4. Ministry of Health *National Oral Health Policy 2022–2030*; Ministry of Health: Nairobi, Kenya, 2022;
5. World Health Organization *Kenya: Oral Health Country Profile*; World Health Organization: Geneva, Switzerland, 2022;
6. Chen, J.; Duangthip, D.; Gao, S.S.; Huang, F.; Anthonappa, R.; Oliveira, B.H.; Turton, B.; Durward, C.; El Tantawi, M.; Attia, D.; et al. Oral Health Policies to Tackle the Burden of Early Childhood Caries: A Review of 14 Countries/Regions. *Front. Oral Health* **2021**, *2*, doi:10.3389/froh.2021.670154.
7. World Health Organization *Uganda: Oral Health Country Profile*; World Health Organization: Geneva, Switzerland, 2022;
8. Northridge, M.E.; Kumar, A.; Kaur, R. Disparities in Access to Oral Health Care. *Annu. Rev. Public Health* **2020**, *41*, 513–535, doi:10.1146/annurev-publhealth-040119-094318.
9. Oral Health in America: Advances and Challenges | NIDCR Available online: https://www.nidcr.nih.gov/research/oralhealthinamerica (accessed on 6 January 2026).
10. Fellows, J.L.; Atchison, K.A.; Chaffin, J.; Chávez, E.M.; Tinanoff, N. Oral Health in America: Implications for Dental Practice. *J. Am. Dent. Assoc.* **2022**, *153*, 601–609, doi:10.1016/j.adaj.2022.04.002.
11. U.S. Department of Health and Human Services Oral Health Strategic Framework, 2014–2017 - , U.S. Department of Health and Human Services Oral Health Coordinating Committee, U.S. Department of Health and Human Services Oral Health Coordinating Committee, Margo R. Adesanya, William Bailey, Donald C. Belcher, Marco Beltran, Tracy Branch, Marcia K. Brand, Edwin M. Craft, Agnes H. Donahue, Bruce A. Dye, Gina Thornton-Evans, Isabel Garcia, Frederick Hyman, Renée Joskow, Arlene M. Lester, Nicholas S. Makrides, Richard J. Manski, Marian Mehegan, Lynn Douglas Mouden, Danielle Nelson, Laurie Norris, Jessica O'Hara, Gail Cherry-Peppers, Timothy L. Ricks, Rochelle Rollins, 2016 Available online: https://journals.sagepub.com/doi/10.1177/003335491613100208 (accessed on 9 February 2026).
12. Quiñonez, C.; Jones, J.A.; Vujicic, M.; Tomar, S.L.; Lee, J.Y. The 2021 Report on Oral Health in America: Directions for the Future of Dental Public Health and the Oral Health Care System. *J. Public Health Dent.* **2022**, *82*, 133–137, doi:10.1111/jphd.12521.
13. Gupta, N.; Vujicic, M.; Yarbrough, C.; Harrison, B. Disparities in Untreated Caries among Children and Adults in the U.S., 2011–2014. *BMC Oral Health* **2018**, *18*, 30, doi:10.1186/s12903-018-0493-7.
14. Cheung, A.; Singhal, S. Towards Equitable Dental Care in Canada: Lessons from the Inception of Medicare. *Int. J. Health Plann. Manage.* **2023**, *38*, 1127–1134, doi:10.1002/hpm.3680.
15. Allison, P.J. Canada's Oral Health and Dental Care Inequalities and the Canadian Dental Care Plan. *Can. J. Public Health.* **2023**, *114*, 530–533, doi:10.17269/s41997-023-00800-6.
16. Rock, L.D.; Akade, G.; Al-Waeli, H.; Allin, S.; Altabtbaei, K.; Ameli, N.; Bassim, C.; Bedos, C.; Benbow, P.; Bhagirath, A.Y.; et al. Canada's First National Oral Health Research Strategy (2024–2030). *J. Dent. Res.* **2025**, *104*, 113–118, doi:10.1177/00220345241299360.

17. Hermosillo, V.H.; Quintero, L.E.; Guerrero, N.D.; Suárez, D.D.S.; Hernández, M.J.A.; Holmgren, C.J. The Implementation and Preliminary Evaluation of an ART Strategy in Mexico: A Country Example. *J. Appl. Oral Sci.* **2009**, *17*, 114–121, doi:https://doi.org/10.1590/S1678-77572009000700019.
18. Pucca, G.A.; Gabriel, M.; de Araujo, M.E.; de Almeida, F.C.S. Ten Years of a National Oral Health Policy in Brazil: Innovation, Boldness, and Numerous Challenges. *J. Dent. Res.* **2015**, *94*, 1333–1337, doi:10.1177/0022034515599979.
19. Santos, L.P. de S.; Lima, A.M.F. de S.; Chaves, S.C.L.; Vilela, D.M.O.C.; Valente, A.P.P.C.; Rossi, T.R.A. Oral Health Policy in Brazil: Changes and Ruptures during the Period 2018-2021. *Ciênc. Saúde Coletiva* **2023**, *28*, 1575–1587, doi:https://doi.org/10.1590/1413-81232023285.14002022EN.
20. Brazil's National Oral Health Policy: An Example for Other Nations Available online: https://www.sciencedaily.com/releases/2015/08/150827154505.htm (accessed on 6 January 2026).
21. World Health Organization *Oral Health Country Profile: Haiti*; World Health Organization: Geneva, Switzerland, 2022;
22. Elamin, A.; Garemo, M.; Gardner, A. Dental Caries and Their Association with Socioeconomic Characteristics, Oral Hygiene Practices and Eating Habits among Preschool Children in Abu Dhabi, United Arab Emirates - the NOPLAS Project. *BMC Oral Health* **2018**, *18*, 104, doi:10.1186/s12903-018-0557-8.
23. Ministry of Health and Prevention *National Strategy for Wellbeing 2031*; Government of the United Arab Emirates: Abu Dhabi, United Arab Emirates, 2018;
24. World Health Organization, Regional Office for the Eastern Mediterranean *Noncommunicable Diseases Country Profile: United Arab Emirates*; World Health Organization Regional Office for the Eastern Mediterranean: Cairo, Egypt, 2022;
25. Ministry of Health *Health Sector Transformation Program: Saudi Vision 2030*; Ministry of Health: Riyadh, Saudi Arabia, 2021;
26. Famurewa, B.A.; Aborisade, A.O.; Dabar, A.M.; Akinsolu, F.T.; El Tantawi, M.; Ezechi, O.C.; Foláyan, M.O. Prevalence and Risk Factors for Early Childhood Caries in North Africa: A Systematic Review and Meta-Analysis. *BMC Oral Health* **2025**, *25*, 1857, doi:10.1186/s12903-025-07419-8.
27. World Health Organization *Libya: Health System Review*; WHO Regional Office for the Eastern Mediterranean: Cairo, Egypt, 2019;
28. Leggett, H.; Vinall-Collier, K.; Csikar, J.; Veronica Ann Douglas, G. Barriers to Prevention in Oral Health Care for English NHS Dental Patients: A Qualitative Study of Views from Key Stakeholders. *BMC Oral Health* **2023**, *23*, 332, doi:10.1186/s12903-023-03030-x.
29. Evans, D.; Mills, I.; Burns, L.; Bryce, M.; Hanks, S. The Dental Workforce Recruitment and Retention Crisis in the UK. *Br. Dent. J.* **2023**, *234*, 573–577, doi:10.1038/s41415-023-5737-5.
30. 'At a Tipping Point': A Comparative Analysis of Oral Health Coverage for Children across Six European Countries: Denmark, Germany, Hungary, Ireland, Scotland, and Spain | BMC Oral Health | Springer Nature Link Available online: https://link.springer.com/article/10.1186/s12903-025-05773-1 (accessed on 9 February 2026).
31. Evaluation of a National Complex Oral Health Improvement Programme: A Population Data Linkage Cohort Study in Scotland | BMJ Open Available online: https://bmjopen.bmj.com/content/10/11/e038116 (accessed on 9 February 2026).
32. World Health Organization *Global Oral Health Status Report: Towards Universal Health Coverage for Oral Health by 2030*; World Health Organization: Geneva, Switzerland, 2022;
33. Palència, L.; Espelt, A.; Cornejo-Ovalle, M.; Borrell, C. Socioeconomic Inequalities in the Use of Dental Care Services in Europe: What Is the Role of Public Coverage? *Community Dent. Oral Epidemiol.* **2014**, *42*, 97–105, doi:10.1111/cdoe.12056.

34. Ziller, S.; Eaton, K.E.; Widström, E. The Healthcare System and the Provision of Oral Healthcare in European Union Member States. Part 1: Germany. *Br. Dent. J.* **2015**, *218*, 239–244, doi:10.1038/sj.bdj.2015.95.
35. Nomura, M. Dental Healthcare Reforms in Germany and Japan: A Comparison of Statutory Health Insurance Policy. *Jpn. Dent. Sci. Rev.* **2008**, *44*, 109–117, doi:10.1016/j.jdsr.2008.06.004.
36. Allin, S.; Farmer, J.; Quiñonez, C.; Peckham, A.; Marchildon, G.; Panteli, D.; Henschke, C.; Fattore, G.; Lamloum, D.; Holden, A.C.L.; et al. Do Health Systems Cover the Mouth? Comparing Dental Care Coverage for Older Adults in Eight Jurisdictions. *Health Policy* **2020**, *124*, 998–1007, doi:10.1016/j.healthpol.2020.06.015.
37. Erdsiek, F.; Waury, D.; Brzoska, P. Oral Health Behaviour in Migrant and Non-Migrant Adults in Germany: The Utilization of Regular Dental Check-Ups. *BMC Oral Health* **2017**, *17*, 84, doi:10.1186/s12903-017-0377-2.
38. Prévention Bucco-Dentaire -La Prévention Dentaire Prend Une Nouvelle Forme Avec « M'T Dents Tous Les Ans ! » | Service Public Available online: https://www.service-public.gouv.fr/particuliers/actualites/A17679 (accessed on 8 January 2026).
39. Nay, O.; Béjean, S.; Benamouzig, D.; Bergeron, H.; Castel, P.; Ventelou, B. Achieving Universal Health Coverage in France: Policy Reforms and the Challenge of Inequalities. *The Lancet* **2016**, *387*, 2236–2249, doi:10.1016/S0140-6736(16)00580-8.
40. Chevreul, K.; Berg Brigham, K.; Durand-Zaleski, I.; Hernandez-Quevedo, C. France: Health System Review. *Health Syst. Transit.* **2015**, *17*, 1–218, xvii.
41. Tubert-Jeannin, S.; Bénézet, L.; Mulliez, A.; Listl, S. The French 100% Santé Reform: Impacts on Dental Care Utilization. *J. Dent. Res.* **2025**, 220345251364167, doi:10.1177/00220345251364167.
42. Ardakani, M.S.Z.; Bayati, M. Global Situation of Oral Health Coverage toward Universal Health Coverage: A Scoping Review. *Prev. Med. Rep.* **2025**, *58*, 103227, doi:10.1016/j.pmedr.2025.103227.
43. Mazevet, M.E.; Garyga, V.; Pitts, N.B.; Pennington, M.W. The Highly Controversial Payment Reform of Dentists in France: Seeking a New Compromise after the 2017 Strike. *Health Policy* **2018**, *122*, 1273–1277, doi:10.1016/j.healthpol.2018.10.001.
44. Bas, A.C.; Azogui-Lévy, S. Evaluation of Children's Participation in a National Dental Programme in France. *Community Dent. Oral Epidemiol.* **2019**, *47*, 291–298, doi:10.1111/cdoe.12456.
45. Bas, A.-C. L'accès aux soins bucco-dentaires dans la réforme 100 % santé : contexte et perspectives. *Santé Publique* **2023**, *35*, 119–124, doi:10.3917/spub.hs1.2023.0119.
46. Ekici, O.; Tengilimoglu, D.; Isik, O. Evaluating the Current Situation of Oral and Dental Healthcare Services in Turkey and Recommending Solutions. *Health Policy Technol.* **2017**, *6*, 368–378, doi:10.1016/j.hlpt.2017.07.006.
47. Doğan, A.; Durukan Köse, S. Oral Health Policy Model for Turkey: How to Deliver Preventive Services? *Front. Health Serv.* **2025**, *5*, doi:10.3389/frhs.2025.1513688.
48. Dental Caries and Associated Factors among Turkish Children and Adults: Findings from the 3rd National Oral Health Survey - Orhan - 2024 - Community Dentistry and Oral Epidemiology - Wiley Online Library Available online: https://onlinelibrary.wiley.com/doi/10.1111/cdoe.12943 (accessed on 9 February 2026).
49. Çakmakoğlu, E.E.; Günay, A. Nationwide Prevalence of Dental Caries in Turkish Children: A Meta-Analysis. *Children* **2025**, *12*, doi:10.3390/children12060777.
50. Topaloglu-Ak, A.; Eden, E.; Frencken, J.E. Managing Dental Caries in Children in Turkey - a Discussion Paper. *BMC Oral Health* **2009**, *9*, 32, doi:10.1186/1472-6831-9-32.
51. FDI World Dental Federation *Thailand Prioritizes Oral Health through Integration into Universal Health Coverage*; FDI World Dental Federation: Geneva, Switzerland, 2019;
52. Tangcharoensathien, V.; Witthayapipopsakul, W.; Panichkriangkrai, W.; Patcharanarumol, W.; Mills, A. Health Systems Development in Thailand: A Solid Platform for Successful Implementation of

Universal Health Coverage. *Lancet Lond. Engl.* **2018**, *391*, 1205–1223, doi:10.1016/S0140-6736(18)30198-3.
53. World Health Organization *Indonesia: Oral Health Country Profile*; World Health Organization: Geneva, Switzerland, 2022;
54. World Health Organization *Sri Lanka: Oral Health Country Profile*; World Health Organization: Geneva, Switzerland, 2022;
55. World Health Organization *Oral Health Country Profile: Nepal*; World Health Organization: Geneva, Switzerland, 2022;
56. Kothia, N.R.; Bommireddy, V.S.; Devaki, T.; Vinnakota, N.R.; Ravoori, S.; Sanikommu, S.; Pachava, S. Assessment of the Status of National Oral Health Policy in India. *Int. J. Health Policy Manag.* **2015**, *4*, 575–581, doi:10.15171/ijhpm.2015.137.
57. Rawat, R.; Aswal, G.S.; Dwivedi, D.; Gurumurthy, V.; Vishwanath, S. Decoding India's National Oral Health Program-an Appraisal of the Barriers to Quality Dental Care. *Int. J. Community Med. Public Health* **2021**, *8*, 458–462, doi:10.18203/2394-6040.ijcmph20205736.
58. Talukdar, R.; Barman, D.; Thakkar, V.; Kanungo, S. Utilization of Dental Care Services among Adult Indian Population: A Meta-Analysis of Evidence from 2011–2022. *Health Promot. Perspect.* **2022**, *12*, 325–335, doi:10.34172/hpp.2022.42.
59. Okamoto, E. Japan's Dental Care Facing Population Aging: How Universal Coverage Responds to the Changing Needs of the Elderly. *Int. J. Environ. Res. Public. Health* **2021**, *18*, 9359, doi:10.3390/ijerph18179359.
60. Saito, M.; Shimazaki, Y.; Fukai, K.; Furuta, M.; Aida, J.; Ando, Y.; Miyazaki, H.; Kambara, M. A Multilevel Analysis of the Importance of Oral Health Instructions for Preventing Tooth Loss: The 8020 Promotion Foundation Study of Japanese Dental Patients. *BMC Oral Health* **2020**, *20*, 328, doi:10.1186/s12903-020-01319-9.
61. CAI He, C.Y. Recent Developments and Future Directions of Oral Healthcare System and Dental Public Health System in China in Light of the Current Global Emergency. *J. Sichuan Univ. Med. Sci. Ed.* **2022**, *53*.
62. World Health Organization *Cambodia: Oral Health Country Profile*; World Health Organization: Geneva, Switzerland, 2022;
